# Optimal control nodes in disease-perturbed networks as targets for combination therapy

**DOI:** 10.1038/s41467-019-10215-y

**Published:** 2019-05-16

**Authors:** Yuxuan Hu, Chia-hui Chen, Yang-yang Ding, Xiao Wen, Bingbo Wang, Lin Gao, Kai Tan

**Affiliations:** 1 School of Computer Science and Technology, Xidian University, Xi’an, Shaanxi 710071 China; 2 Division of Oncology and Center for Childhood Cancer Research, 4004 CTRB, Children’s Hospital of Philadelphia, 3501 Civic Center Boulevard, Philadelphia, PA 19104 USA; 3 Department of Biomedical and Health Informatics, Children’s Hospital of Philadelphia, Philadelphia, PA 19104 USA; 4 Department of Pediatrics, Perelman School of Medicine, University of Pennsylvania, Philadelphia, PA 19104 USA

**Keywords:** Computational biology and bioinformatics, Cellular signalling networks, Computational models, Data integration, Gene regulatory networks

## Abstract

Most combination therapies are developed based on targets of existing drugs, which only represent a small portion of the human proteome. We introduce a network controllability-based method, OptiCon, for de novo identification of synergistic regulators as candidates for combination therapy. These regulators jointly exert maximal control over deregulated genes but minimal control over unperturbed genes in a disease. Using data from three cancer types, we show that 68% of predicted regulators are either known drug targets or have a critical role in cancer development. Predicted regulators are depleted for known proteins associated with side effects. Predicted synergy is supported by disease-specific and clinically relevant synthetic lethal interactions and experimental validation. A significant portion of genes regulated by synergistic regulators participate in dense interactions between co-regulated subnetworks and contribute to therapy resistance. OptiCon represents a general framework for systemic and de novo identification of synergistic regulators underlying a cellular state transition.

## Abstract (web summary)

Synergistic interactions may arise between regulators in complex molecular networks. Here, the authors develop OptiCon, a computational method for de novo identification of synergistic key regulators and investigate their potential roles as candidate targets for combination therapy.

## Introduction

Complex diseases arise from multiple deregulated pathways. Given the diversity of biological processes underlying pathogenesis, monotherapy is unlikely to be curative as single agents. There are also multiple redundancies or alternative pathways that may be activated in response to the inhibition of a pathway, which is often due to the crosstalk among pathways. Crosstalk promotes the emergence of resistant cells under the selective pressure of a targeted agent, resulting in drug resistance and clinical relapse^1^.

Combination therapy, by exerting effects on more than one pathogenic pathway, is an effective strategy to combat drug resistance and disease heterogeneity. Ever since the first success with combination chemotherapy on childhood acute lymphoblastic leukemia^2^, combination therapy has been developed to treat many complex diseases, including cancers^3^, infectious diseases^4^, and neurodegenerative diseases^5^. Traditionally, development of combination therapy has been pursued one agent at a time, with the investigational drug(s) tested for efficacy in add-on trials. In recent years, a number of computational methods have been developed for discovering combination therapeutic targets^6,7^, including network-based approaches^8–10^. These network-based approaches are motivated by the observation that multiple disease relevant genes, rather than a single gene, often interact within a complex gene network, resulting in a disease phenotype and drug resistance. A representative network-based method is the ranking-system of anti-cancer synergy (RACS) algorithm^9^. It predicts synergistic anticancer drugs using drug-treated transcriptome profiles and protein–protein interaction network and KEGG pathways. DrugComboRanker^10^ is another representative network-based method to identify known drug combinations using drug-treated gene expression profiles. The majority of existing methods focused on identifying synergistic combinations of existing drugs. However, targets of existing drugs are limited to a small group of proteins. There are only 667 human proteins as targets of approved drugs^11^ as of 2016. Thus, a large number of genes and their combinations remain unexplored as potential combinatorial therapeutic targets. In addition, requirement of drug-treated experimental data as input limits the applicability of the methods. An exception is the virtual inference of protein activity by enriched regulon analysis (VIPER) algorithm^12^. VIPER uses the principle in the Master Regulator Inference algorithm^13^ to identify synergistic master regulators for phenotypic transitions, which are considered as candidate combinatorial targets in the context of drug discovery.

Recently, network controllability theory has emerged as an attractive theoretical framework for developing network-based algorithms^14–16^. The theory provides a mathematically validated framework for identifying a minimal set of driver nodes that can drive the transition of the network between any two states. Controllability theory has been successfully applied to predict drug targets or driver molecules using human metabolic networks^17^, protein–protein interaction networks^18^ and transcriptional regulatory networks^19^. However, these studies only applied controllability theory to binary networks without considering the activities of the genes or levels of metabolites, which underestimates the ability of a node to control the dynamics of the network. As a result, previous studies typically identify a large portion of the input network as driver nodes due to the sparsity and degree heterogeneity of molecular networks^14^. The large number of driver nodes presents a daunting challenge for candidate prioritization and experimental follow-up.

Here, we describe the Optimal Control Node (OptiCon) algorithm, a general framework for systematic and de novo identification of synergistic key regulators in a gene regulatory network. By using gene expression as a constraint in the standard network controllability framework, OptiCon first identifies a set of optimal control nodes (OCNs) in a disease-perturbed gene regulatory network. The identified OCNs exert maximal control over deregulated pathways but minimal control over pathways that are not perturbed by the disease. Next, using a synergy score that combines both genetic mutation and gene functional interaction information, OptiCon identifies a set of synergistic OCNs as key regulators in the disease-perturbed network, which can serve as candidate targets for combination therapy.

## Results

### Use of network controllability for identifying drug targets

**Fig. 1:**
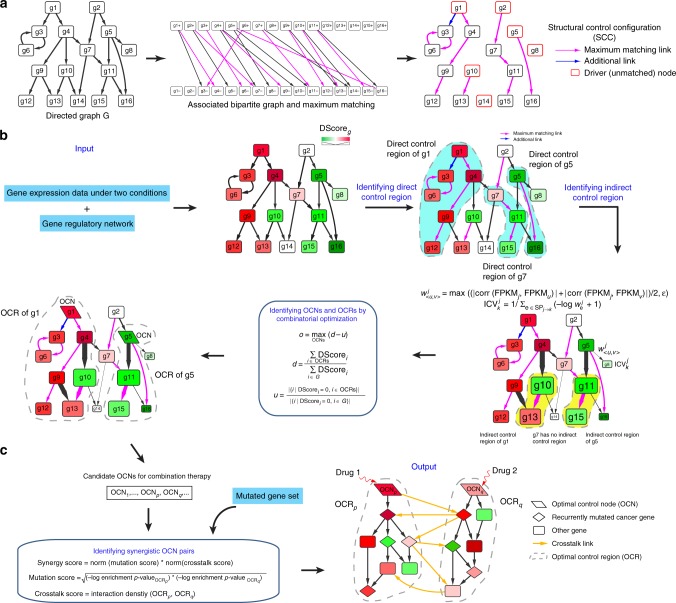
Network controllability theory and overview of the OptiCon algorithm. **a** Identification of driver nodes in a directed graph based on structural controllability theory. By converting a directed graph *G* into a bipartite graph, a structural control configuration (SCC) of the network can be identified by finding a maximum matching in the bipartite graph. SCC consists of a spanning subgraph with the same node set as *G*, a maximum matching of *G* and an additional link set. The matching links divide the network *G* into several elementary paths and elementary cycles. The additional links transmit signals from the elementary paths to the elementary cycles. The unmatched nodes (in red) comprise the minimal set of driver nodes that can control the dynamics of the entire network from any initial state to any desired final state. **b**, **c** Overview of OptiCon for identifying optimal control nodes (OCNs) and their synergistic combinations. **b** Using gene expression data under two conditions (e.g., diseased vs. healthy) and a directed gene regulatory network as inputs to OptiCon, a deregulation score (DScore)-weighted network can be obtained. The control region of a gene in the DScore-weighted network consists of a direct control region and an indirect control region. Direct control region (highlighted in cyan) of a gene is identified by finding the structural control configuration of the network. Indirect control region (highlighted in yellow) is identified by using the indirect control value (ICV) and a shortest path (SP) search procedure. The candidate OCNs for combination therapy can be identified using a combinatorial optimization procedure. For clarity, only the control regions of *g*1, *g*5 and *g*7 are shown instead of all genes. *o*, *d*, and *u*, the optimal influence, desired influence and undesired influence by the candidate OCNs, respectively. **c** Identification of synergistic OCN pairs using synergy score. The synergy score consists of two parts. The mutation score measures the enrichment of recurrently mutated cancer genes in the optimal control region (OCR) of each OCN. The crosstalk score measures the interaction density between genes in the OCRs of the two OCNs under consideration. Norm, min-max normalization

In network controllability theory, a structural control configuration (SCC) of a network characterizes a topological skeleton for controlling the dynamics of the network from any initial state to any desired final state using a minimal set of driver nodes^14,20^. Given a gene regulatory network *G*, its SCC can be identified by finding a maximum matching in the bipartite graph representing *G* (Fig. 1a; Supplementary Methods). SCC is a spanning subnetwork of the network *G* consisting of the same node set as *G*, a maximum matching of *G* and an additional link set^20^ (Fig. 1a, right panel). The matching links divide the original network *G* into several elementary paths and elementary cycles. The additional link set includes remaining links (i.e., directed edges in the original network excluding edges in elementary paths and elementary cycles) that start at the nodes (excluding the terminal nodes) of the elementary paths and end at the nodes of the elementary cycles. As an example in Fig. 1a, there are 10 maximum matching links that divide the network *G* into six elementary paths (i.e., $\left\{ {g1 \to g4 \to g9 \to g12} \right\}$, $\left\{ {g10 \to g13} \right\}$, $\left\{ {g14} \right\}$, $\left\{ {g2 \to g7 \to g11 \to g15} \right\}$, $\left\{ {g5 \to g16} \right\}$, and $\left\{ {g8} \right\}$) and one elementary cycle (i.e., $\left\{ {g3 \to g6 \to g3} \right\}$). There is also one additional link $\left\{ {g1 \to g3} \right\}$ that starts at node *g*1 in the elementary path $\left\{ {g1 \to g4 \to g9 \to g12} \right\}$ and ends at node *g*3 in the elementary cycle $\left\{ {g3 \to g6 \to g3} \right\}$. The unmatched nodes (i.e., *g*1, *g*10, *g*14, *g*2, *g*5, and *g*8) comprise the minimal set of driver nodes that can control the dynamics of the entire network^14^. These driver nodes thus represent candidate drug targets because of their ability to guide the network from a diseased state to a healthy state. However, when applying this basic framework to a real, large-scale gene regulatory network comprising 5959 genes and 108,281 regulatory links (Supplementary Data File 1), the minimal set of driver nodes consists of 2754 genes that make up 46% of the input network. The large number of driver nodes is due to the sparsity and degree heterogeneity of the gene regulatory network^14^, which are common features of molecular networks^21^. This result suggests that it is not practical to apply the basic theoretic framework to identify candidate drug targets because of the high therapeutic cost (i.e., a large number of genes need to be targeted). Moreover, some of the driver nodes identified above control genes that are not deregulated under the diseased condition (e.g., *g*2 and *g*14 in Fig. 1b). It is not necessary to control these genes because doing so may cause side effect. These two challenges motivated us to develop the OptiCon (Optimal Control Node) algorithm for identifying a set of OCNs that maximize the control over the deregulated part of the gene network and minimize the control over the unperturbed part of the network. The set of OCNs are searched from all genes in the network and can be considered as candidate drug targets.

### Finding OCNs in a gene regulatory network

A schematic overview of OptiCon is shown in Fig. 1b, c. For each gene in the network, we first define its control region to quantify its ability to control the dynamics of the gene regulatory network. The control region consists of two parts: directly and indirectly controlled regions. Based on structural controllability theory, a gene *i* can fully control the dynamics of its downstream genes (including itself) located in the SCC of the network^20^. These genes are considered directly controllable by gene *i*. Because the directly controllable deregulated genes can influence their downstream genes, gene *i* can also have indirect control over additional genes. Related work on predicting gene functions using network data has shown significant benefits of considering indirect network connections^22,23^. We therefore identify the indirect control region using expression correlation and a shortest path search algorithm (see “Methods”).

Once the control region is defined for each gene in the network, we proceed to identify the OCNs. Because the SCC of a network is not unique (Supplementary Methods), the control region of a gene is not unique. Thus, we should consider a large number of SCCs of a given network in order to identify the optimal control region (OCR) for an OCN. We formulate the identification of OCNs and their OCRs as a combinatorial optimization problem. The objective function consists of three variables: *o*, *d* and *u*, representing the optimal influence, desired influence and undesired influence by the candidate OCNs, respectively. The desired influence *d* is defined as the fraction of the amount of deregulation (i.e., DScore, “Methods” section) that can be controlled by OCNs, while the undesired influence *u* is defined as the fraction of controllable genes that are not deregulated in disease. The objective is to identify a set of OCNs that maximizes the optimal influence $o = d - u$. This optimization problem can be solved using a greedy search algorithm (Supplementary Methods). By comparing to a null distribution of OCN occurrence frequency, a set of OCNs with a given false discovery rate (FDR) cutoff can be identified (“Methods” section). In this paper, we used 0.05 as the FDR cutoff.

### Synergistic OCNs as candidate targets

Although OptiCon identifies a set of OCNs, it does not explicitly reveal which pairs of OCNs are synergistic. For this purpose, we introduce a metric to quantify the synergy between a pair of identified OCNs (Fig. 1c). The synergy score consists of two parts, a mutation score and a crosstalk score. The mutation score measures the enrichment of recurrently mutated cancer genes in the OCR of each OCN. The crosstalk score measures the density of functional interactions between genes in the OCRs of the two OCNs. By comparing the observed synergy score to a null distribution of expected synergy score generated based on 10 million randomly selected gene pairs from the input network, OCN pairs with significantly high synergy scores (empirical *p*-value < 0.05) can be identified.

### Performance evaluation

We first constructed a high-quality gene regulatory network (Supplementary Data File 1) by combining entries from three manually curated pathway databases, Reactome^24^, Kyoto Encyclopedia of Genes and Genomes (KEGG)^25^, and NCI-Nature Pathway Interaction Database^26^. We then combined the network with gene expression data from three cancer types, hepatocellular carcinoma (HCC), lung adenocarcinoma (LUAD), and breast invasive carcinoma (BRCA) to construct three deregulated networks as the input to the algorithms. As baseline comparisons, we compared OptiCon to a method that is also based on network controllability theory, TargetControl^16^ and a method that is based on the in-degree and out-degree distribution of a network (see “Methods” for details). Similar to OptiCon, TargetControl identifies a set of nodes that efficiently control a pre-selected set of other nodes in a network. TargetControl and the degree-based method do not predict synergistic regulators. We therefore compared OptiCon to two additional methods that do predict synergistic regulators, Virtual Inference of Protein activity from Enriched Regulon Analysis (VIPER)^12^ and RACS^9^. VIPER uses the Master Regulator Inference algorithm^13^ to identify synergistic master regulators based on gene expression data and a gene regulatory network. RACS is a semi-supervised learning method that combines drug pharmacological characteristics, drug-targeted networks and transcriptomic profiles to identify potential synergistic combinations of existing cancer drugs.

**Fig. 2:**
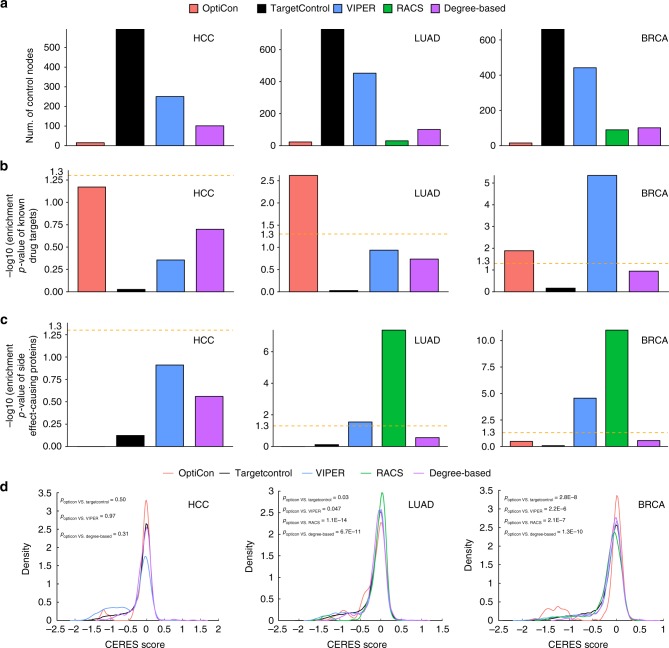
Performance assessment of predicted regulators. **a** Number of key regulators predicted by OptiCon, TargetControl, VIPER, RACS and a degree-based method. HCC, hepatocellular carcinoma; LUAD, lung adenocarcinoma; BRCA, breast invasive carcinoma. **b** Enrichment *p*-values of known cancer drug targets documented in the Therapeutic Target Database. RACS is not included in the comparison because RACS predictions are based on known cancer drug targets. **c** Enrichment *p*-values of side effect-causing proteins. No overlap with OptiCon predictions in HCC and LUAD. **d** Distribution of CERES scores of identified key regulators. *P*-values in **d** were computed using one-sided Kolmogorov–Smirnov test. The rest of the *p*-values were computed using hypergeometric distribution. Yellow dashed line, enrichment *p*-value of 0.05

OptiCon identified 15, 23 and 15 OCNs for HCC, LUAD and BRCA, respectively. In contrast, TargetControl and VIPER identified much larger numbers of control nodes, 593, 727, and 660 in HCC, LUAD, and BRCA by TargetControl and 250, 453, and 442 by VIPER (Fig. 2a). Such large numbers of control nodes represent a formidable challenge for follow-up studies, in particular for identifying synergistic gene pairs.

We next used three additional sets of orthogonal data to further evaluate the predicted regulators by the methods, including known cancer drug targets, cancer vulnerability genes identified by loss-of-function screening, and known proteins that contribute to therapeutic side effect. First, using the Therapeutic Target Database^27^, we found that predictions by OptiCon have higher enrichment for known cancer drug targets than predictions by other methods for HCC, LUAD, and BRCA (hypergeometric test *p*-values = 0.067, 0.002 and 0.013, respectively, Fig. 2b). Second, Meyers et al.^28^ recently conducted a large-scale screening for cancer vulnerability genes using the CRISPR-Cas9 system. Using their data, we found that predictions by OptiCon are more enriched for essential genes than predictions by other methods in LUAD and BRCA (Kolmogorov-Smirnov test *p*-values < 0.05, Fig. 2d). Finally, to evaluate potential side effect of targeting the predicted regulators, we used a list of 237 proteins that are reported to be associated with treatment side effects in various diseases from a published study^29^ (Supplementary Data File 9). We found that OptiCon predictions are depleted of side effect-causing proteins in all three cancers studied. In contrast, targets predicted by VIPER and RACS are enriched for side-effect causing proteins in LUAD and BRCA (Fig. 2c).

**Fig. 3:**
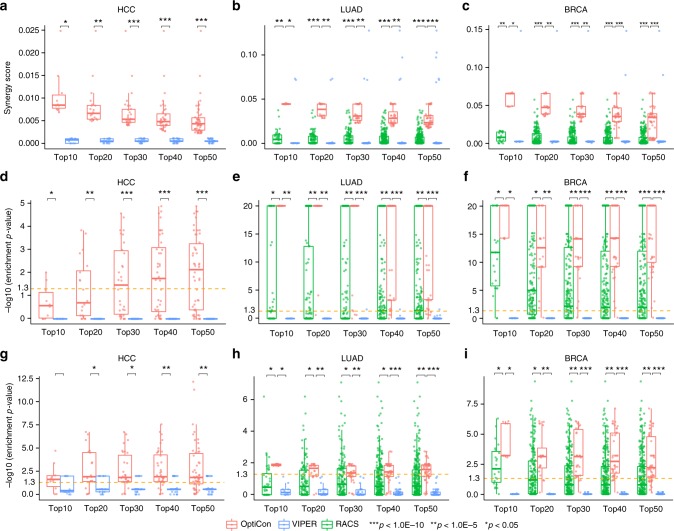
Performance assessment of predicted synergistic interactions. Top ranked synergistic gene pairs, predicted by OptiCon, VIPER, and RACS are evaluated using synergy score and enrichment of synthetic lethal interactions. **a**–**c** Synergy scores of predicted gene pairs. **d**–**f** Enrichment *p*-values of experimentally derived synthetic lethal interactions and **g**–**i** clinically relevant synthetic lethal interactions between the subnetworks targeted by predicted gene pairs. *p*-values for comparing methods in all panels were computed using one-sided Wilcoxon test. Enrichment *p*-values in panels (**d**–**i**) were computed using hypergeometric distribution. Lower, middle and upper lines of boxplots represent first quartile, median, and third quartile respectively. Lower and upper whiskers represent smallest values within 1.5 times interquartile range below Q1 and above Q3, respectively. Yellow dashed line, enrichment *p*-value of 0.05. HCC hepatocellular carcinoma, LUAD lung adenocarcinoma, BRCA breast invasive carcinoma. No synergistic pairs were predicted by RACS for HCC

Using OptiCon, we identified 77, 192, and 63 synergistic gene pairs (i.e., OCN pairs) for HCC, LUAD and BRCA, respectively. In contrast, VIPER identified a larger number of synergistic gene pairs, 283 for HCC, 894 for LUAD, and 1554 for BRCA. Due to the limited number of existing cancer drugs, RACS only identified 55 and 33 synergistic drug pairs for LUAD and BRCA and HCC was not studied by the authors of RACS. We found that gene pairs predicted by OptiCon have significantly higher synergy scores than those predicted by either VIPER or RACS (Wilcoxon test *p*-values < 0.05, Fig. 3a–c).

Functional synergy between two regulators is mediated through interactions among their downstream genes. We therefore further evaluated the performance of the methods using synthetic lethal interactions. We downloaded experimentally derived cancer-type-specific synthetic lethal interactions curated in the Synthetic Lethality Database (SynLethDB, Supplementary Data File 3)^30^ and the study by Shen et al.^31^. Clinically relevant synthetic lethal interactions were downloaded from a recent study by Lee et al.^32^. Compared to VIPER and RACS, we found that OCRs controlled by OCN pairs predicted by OptiCon have significantly higher enrichment (Wilcoxon test *p*-values < 0.05) for both experimentally derived (Fig. 3d–f) and clinically relevant synthetic lethal interactions (Fig. 3g–i).

Taken together, these results demonstrate significant improvement of OptiCon over three state-of-the-art methods, TargetControl, VIPER and RACS. Moreover, the enrichment of synthetic lethal interactions among genes downstream of the predicted OCN pairs implies that targeting them can not only control oncogenic pathways but also their functional buffering pathways.

### Case study 1: liver cancer

HCC is the most common form of liver cancer. Using OptiCon, we identified 15 OCNs (Supplementary Data File 4). Among them, three (NCSTN, APH1A and MAPKAPK2) are known drug targets for HCC (hypergeometric test *p*-value = 0.067). Seven additional OCNs have been shown to play an important role in the progression or metastasis of HCC (Supplementary Notes). The remaining five OCNs are novel predictions.

**Fig. 4:**
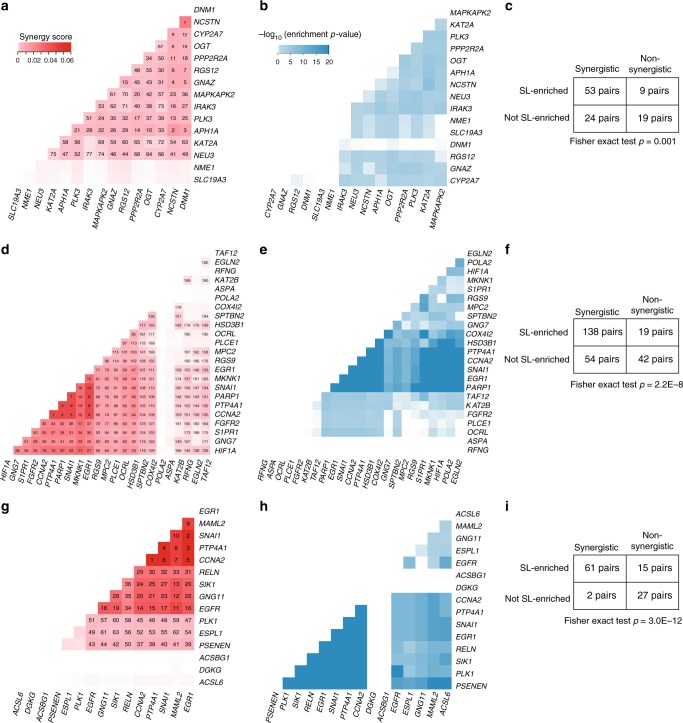
Synergistic optimal control nodes discovered in three cancer types. **a**, **d**, **g** 77, 192, and 63 significantly synergistic optimal control node (OCN) pairs (Benjamini–Hochberg adjusted empirical *p*-values < 0.05) identified in hepatocellular carcinoma (HCC), lung adenocarcinoma (LUAD) and breast invasive carcinoma (BRCA), respectively. Shade of red in the heat map is proportional to the synergy score. Numbers represent the ranks of the identified synergistic pairs. **b**, **e**, **h** Experimentally derived cancer-type-specific synthetic lethal interactions are significantly enriched between optimal control regions (OCRs) of 53 (69%), 138 (72%), and 61 (97%) synergistic OCN pairs identified in HCC, LUAD and BRCA, respectively (Benjamini–Hochberg adjusted hypergeometric *p*-values < 0.05). Shade of blue in the heat map is inversely proportional to the enrichment *p*-values. **c**, **f**, **i** Contingency tables and corresponding Fisher’s exact test *p*-values are shown for HCC, LUAD and BRCA, respectively, indicating that known synthetic lethal interactions (SL) are more enriched between OCRs of synergistic OCN pairs than non-synergistic OCN pairs

We identified 77 synergistic OCN pairs as candidate targets for combination therapy (empirical *p*-values < 0.05, Fig. 4a, Supplementary Data File 5). As supporting evidence for the predicted synergy, we found that both experimentally derived^30,31^ and clinically relevant^32^ synthetic lethal interactions are more enriched between OCRs of synergistic OCNs than non-synergistic OCNs (Fisher’s exact test *p*-values = 0.001 and 1.1E–9, Fig. 4c and Supplementary Fig. 4b). In total, OCRs of 53 (69%) and 52 (68%) synergistic OCN pairs are enriched for known experimental and clinically relevant synthetic lethal interactions between them, respectively (Fig. 4b and Supplementary Fig. 4a).

To further evaluate the clinical relevance of the predicted OCN pairs, we performed survival analysis using the predicted OCN pairs as co-variates in the Cox proportional hazards model. Overall, we found that 56% of the synergistic OCN pairs have significant interaction (Cox *p*-value < 0.1) that is associated with survival time of liver cancer patients (Supplementary Data File 5).

**Fig. 5:**
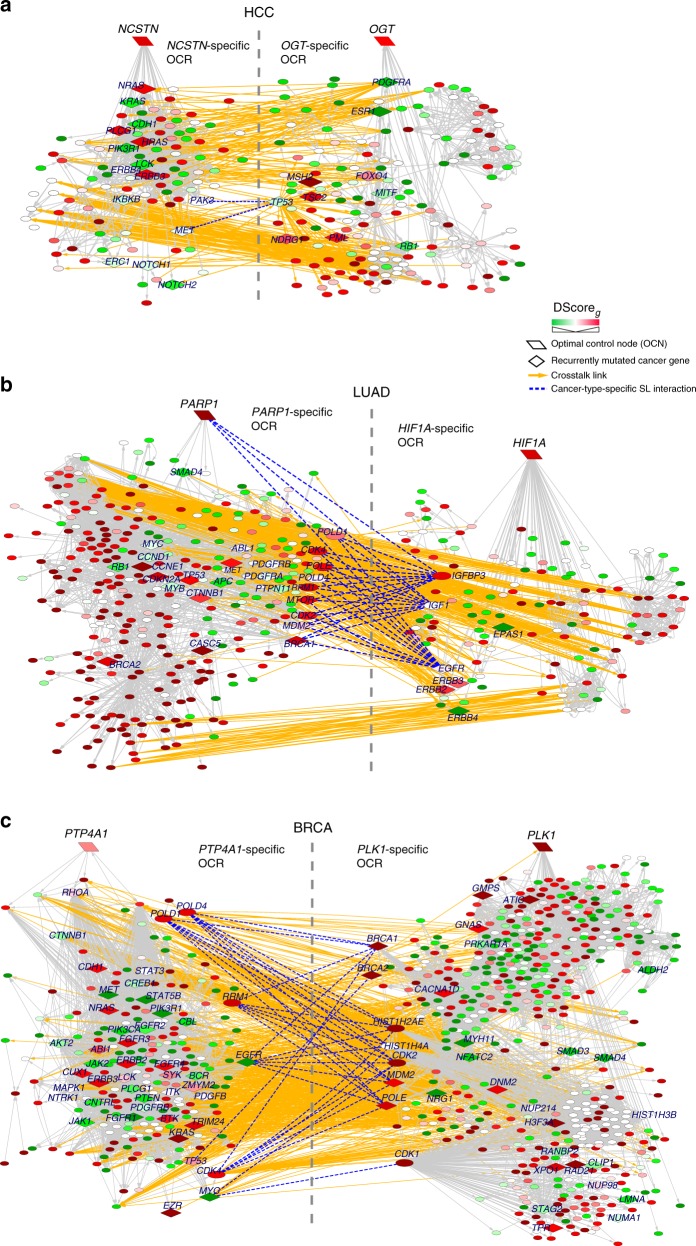
Representative synergistic OCN pairs predicted for three cancer types. **a** Representative synergistic optimal control node (OCN) pair predicted for liver cancer, *NCSTN* and *OGT*, and crosstalk links between their specific optimal control regions (OCRs). For clarity, only genes that are known to be recurrently mutated in liver cancer and/or involved in known synthetic lethal (SL) interactions are labelled. Shade of a node represents the deregulation score (DScore) of the corresponding gene. Red, up-regulation in cancer samples; green, down-regulation in cancer samples. Yellow links, crosstalk links between the OCRs of the two OCNs. Blue dashed lines, known cancer-type-specific synthetic lethal interactions. **b** Representative synergistic OCN pair predicted for lung cancer, *PARP1* and *HIF1A*, and crosstalk links between their specific OCRs. **c** Representative synergistic OCN pair predicted for breast cancer, *PLK1* and *PTP4A1*, and crosstalk links between their specific OCRs

An interesting OCN pair consists of *NCSTN* and *OGT* (Fig. 5a). *NCSTN* encodes a subunit of the gamma-secretase complex in the Notch signaling pathway, whose activation promotes the formation of HCC in vivo^33^. Knockdown of *NCSTN* significantly inhibited HCC cell growth *i*n vitro^34^. *OGT* encodes an enzyme for the addition of O-linked N-acetylglucosamine (O-GlcNAc) to protein substrates. Knockdown of *OGT* was demonstrated to reduce the survival, migrating and invasive ability of HCC cell in vitro and inhibit HCC tumorigenesis and metastasis in vivo^35^.

### Case study 2: lung cancer

LUAD is the most common form of non-small cell lung cancer. Using OptiCon, we identified 23 OCNs (Supplementary Data File 4). Among them, six (PARP1, HIF1A, S1PR1, FGFR2, MKNK1, and PTP4A1) are known drug targets for LUAD (hypergeometric test *p*-value = 0.002). Seven additional OCNs have been shown to play a role in LUAD progression (Supplementary Notes). The remaining ten OCNs are novel predictions.

We predicted 192 synergistic OCN pairs (empirical *p*-values < 0.05) as candidates for combination therapy (Fig. 4d, Supplementary Data File 5). As supporting evidence for the predicted synergy among OCNs, we found that both experimentally derived and clinically relevant synthetic lethal interactions are more enriched between OCRs of synergistic OCNs than non-synergistic OCNs (Fisher’s exact test *p*-values = 2.2E–8 and 2.0E–9, Fig. 4f and Supplementary Fig. 4d). In total, OCRs of 138 (72%) and 89 (46%) synergistic OCN pairs are enriched for known lung cancer-specific and clinically relevant synthetic lethal interactions between them, respectively (Fig. 4e and Supplementary Fig. 4c). Furthermore, we found that 55% of the synergistic OCN pairs have significant interaction (Cox *p*-value < 0.1) that is associated with survival time of lung cancer patients (Supplementary Data File 5). An example pair consists of *PARP1* and *HIF1A* (Fig. 5b). PARP1 inhibition results in accumulation of DNA double strand breaks and a potent anti-proliferation effect on *ERCC1* or *PTEN*-deficient LUAD cells since these cells have DNA damage repair defects^36^. Functional interactions between *HIF1A* and DNA-damage response pathway have been reported^37^. In addition, *HIF1A* was reported to confer protection against chemotherapy-induced DNA damage, resulting in drug resistance^38^. Our result showed that *PARP1* and *HIF1A* have 41 known lung cancer-specific (hypergeometric test *p*-value < 1.0E–20) and 30 clinically relevant (*p*-value = 0.04) synthetic lethal interactions between their OCRs. Taken together, these results strongly suggest that PARP1 and HIF1A are effective combinatorial therapeutic targets for lung cancer.

### Case study 3: breast cancer

Among the 15 OCNs predicted by OptiCon (Supplementary Data File 4), four (PLK1, EGFR, PTP4A1 and PSENEN) are known drug targets for BRCA (hypergeometric test *p*-value = 0.013). Eight additional OCNs also play critical roles in BRCA. We also identified three novel key regulators for BRCA, such as DGKG, a member of the diacylglycerol kinase family (DGKs). DGKs can modulate several oncogenic signaling pathways (e.g., *RAS* and the extracellular signal-regulated kinase cascade) and are considered important regulators of cancer progression^39^. A recent study also suggests that *DGKG* is a tumor suppressor in colorectal cancer^40^.

We identified 63 synergistic OCN pairs (empirical *p*-values < 0.05) as candidates for combination therapy (Fig. 4g, Supplementary Data File 5). Similar to liver and lung cancers, we also found that both experimentally derived and clinically relevant synthetic lethal interactions are more enriched between OCRs of synergistic OCNs than non-synergistic OCNs (Fisher’s exact test *p*-values = 3.0E–12 and 1.7E–4, Fig. 4i and Supplementary Fig. 4f). In total, OCRs of 61 (97%) and 49 (78%) synergistic OCN pairs are enriched for known breast cancer-specific and clinically relevant synthetic lethal interactions between them, respectively (Fig. 4h and Supplementary Fig. 4e). Furthermore, we found that 63% of the synergistic OCN pairs have significant interaction (Cox *p*-value < 0.1) that is associated with survival time of breast cancer patients (Supplementary Data File 5).

Two known drug targets, PLK1 and PTP4A1, represent a novel synergistic gene pair (Fig. 5c). There are 55 known breast cancer-specific (hypergeometric test *p*-value < 1.0E–20) and 66 clinically relevant (*p*-value = 0.1) synthetic lethal interactions between their OCRs. Based on these results together with their known inhibitory effects on cancer cell proliferation and metastasis^41,42^, combined treatment with both PLK1 and PTP4A1 inhibitors could be an effective therapy for metastatic breast cancer.

### Experimental validation of predicted synergistic OCN pairs

**Fig. 6:**
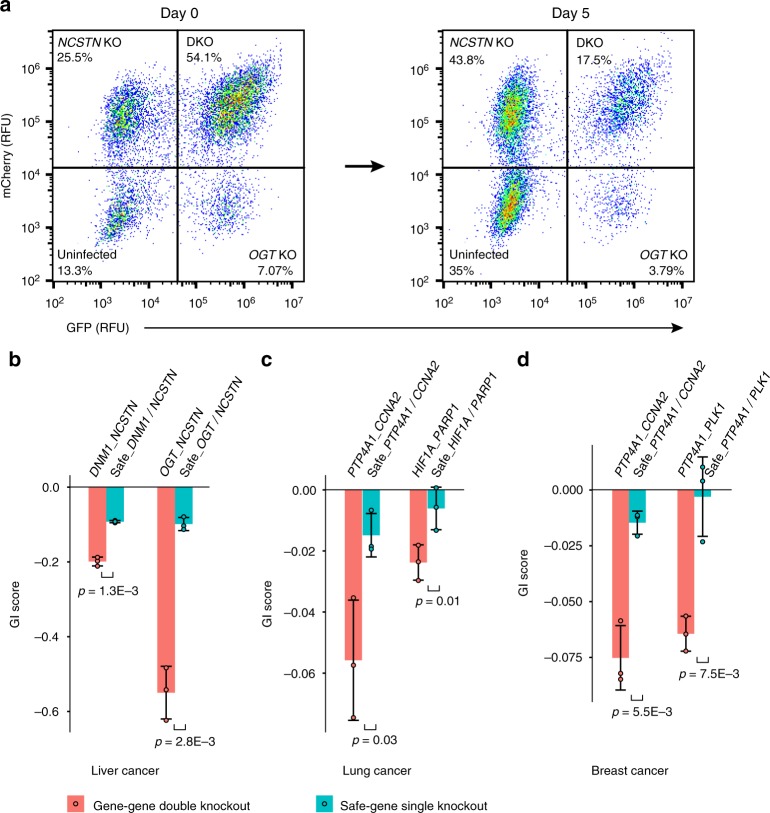
Experimental validation of predicted synergistic pairs. **a** Representative FACS plot of cells infected with lentiviruses expressing *OGT*-targeting (GFP) and *NCSTN*-targeting (mCherry) sgRNAs. Day 0 (4 days post-transduction) and Day 5 data are shown. Value in each quadrant indicates the percentage of cells expressing a given reporter in the culture. The growth phenotype is calculated by measuring the relative depletion of the single-infected and double-infected cells between the start and the end of the growth assay. KO, single knockout. DKO, double knockout. RFU, relative fluorescence unit. Synergistic optimal control nodes validated in liver cancer (**b**), lung cancer (**c**), and breast cancer (**d**). Safe indicates non-targeting control sgRNA. GI score, genetic interaction score. Data represent mean ± s.d. from three replicate cultures. *P*-values were computed using one-sided *t*-test. Source data are provided as a Source Data file

To experimentally test the predicted synergistic regulators, we used a CRISPR-based knockout growth assay^43^. The protocol uses fluorescent reporter genes to track the growth of cells carrying single or double knockout constructs (Fig. 6a; Supplementary Fig. 5). The growth phenotypes of the mutant cells are calculated as the relative depletion of fluorescent signals compared to wild type cells. A genetic interaction score (GI score, either of two-gene knockout or of gene-safe knockout, see “Methods” for details) is determined by comparing their observed and expected growth phenotypes. Finally, synergy between two genes is determined by comparing their GI score to those of gene-safe knockouts. We tested two predicted OCN pairs in each cancer type, including *NCSTN* and *DNM1*, and *NCSTN* and *OGT* in liver cancer; *CCNA2* and *PTP4A1*, and *HIF1A* and *PARP1* in lung cancer; and *CCNA2* and *PTP4A1*, and *PLK1* and *PTP4A1* in breast cancer. For all six OCN pairs tested, we demonstrated that targeting them has a significantly synergistic anti-tumor effect than targeting each gene individually (Student’s *t*-test *p*-values < 0.05, Fig. 6b–d).

### Crosstalk genes play an important role in therapy resistance

**Fig. 7:**
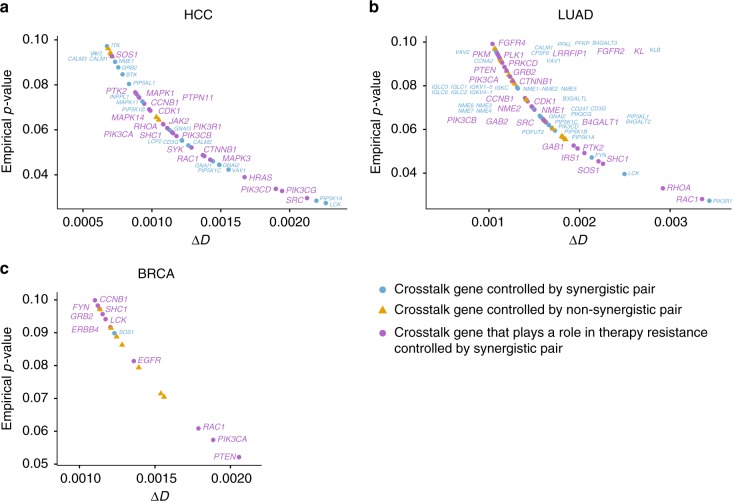
Crosstalk genes play an important role in therapy resistance. Effect of crosstalk genes on the interaction density between optimal control regions (OCRs) of an optimal control node (OCN) pair is quantified as the decrease in interaction density of two OCRs after a crosstalk gene is removed from the OCR, herein termed Δ*D*. For each cancer type, crosstalk genes with significant Δ*D* (empirical *p*-value < 0.1) are shown. Empirical *p*-value was calculated using a null distribution of crosstalk genes controlled by one million randomly selected gene pairs from the input network. Gene symbols are ordered from top to bottom in ascending statistical significance. 21 (50%), 26 (41%), and 10 (91%) of the crosstalk genes controlled by synergistic OCN pairs (magenta dots) have a known role in drug resistance in liver cancer (**a**), lung cancer (**b**) and breast cancer (**c**), respectively (Supplementary Data File 6). In contrast, 0, 7 (28%) and 2 (17%) of the crosstalk genes controlled by non-synergistic OCN pairs (yellow triangles) have a known role in drug resistance in the respective cancer types. HCC hepatocellular carcinoma, LUAD lung adenocarcinoma, BRCA breast invasive carcinoma

In order to better understand the interactions between synergistic OCNs, we examined the OCRs of the synergistic OCNs. Specifically, we focused on genes that are involved in the interactions bridging two OCRs, hereby termed crosstalk genes. Since high interaction density among genes increases the chance of pathway rewiring, which has been suggested as a potential mechanism for the development of drug resistance^1^, we ranked all crosstalk genes in each cancer type based on their effect on the interaction density between two OCRs. The effect is quantified as the decrease in interaction density of two OCRs after a crosstalk gene is removed from an OCR, hereby termed Δ*D*. We conducted a comprehensive literature search of the crosstalk genes with significant Δ*D* (empirical *p*-value < 0.1) controlled by synergistic OCNs. We found that 21 (50%), 26 (41%), and 10 (91%) of these genes (Fig. 7) are known to play important roles in drug resistance in the treatment for liver, lung and breast cancers, respectively (Supplementary Data File 6). For example, *RAC1*, a top-ranked crosstalk gene in all three cancer types, was demonstrated to contribute to multidrug resistance in liver cancer^44^, gefitinib resistance in non-small-cell lung cancer^45^ and trastuzumab resistance in breast cancer^46^. In contrast to genes controlled by synergistic OCNs, only 0, 7 (28%) and 2 (17%) of crosstalk genes controlled by non-synergistic OCN pairs have a known role in drug resistance in the three respective cancer types (Supplementary Data File 6).

## Discussion

We introduce a network controllability-based method, OptiCon, to discover synergistic key regulators as candidate targets for combination therapy. Directly searching for synergistic gene pairs is not a good strategy because the combinatorial search space is huge and thus the burden of multi-testing correction is large. OptiCon tackles this problem in two steps. First, it identifies a set of OCNs. Unlike previous network controllability-based approaches^17–19^, OptiCon considers gene activities as additional constraints when searching for OCNs. The identified control nodes exert maximal control over deregulated genes but minimal control over unperturbed genes in a disease, which could minimize drug side effect when the control nodes are targeted by drugs. Using literature evidence, including known cancer drug targets^27^, cancer essentiality genes^28^, a database of mutated cancer genes^47^, we demonstrate that the identified OCNs are strongly supported by multiple lines of literature evidence. In the second step, OptiCon uses the synergy score to identify significantly synergistic OCN pairs. The synergy score is motivated by our current understanding of drug resistance mechanisms and captures both recurrently mutated genes and crosstalk between the pathways controlled by a pair of OCNs. Synergistic pairs predicted by OptiCon is supported by synthetic lethal interactions and experimental testing using a CRISPR-Cas9-based growth assays. To further evaluate if the gene-gene synergy predicted by OptiCon is a valid approach to nominate targets of synergistic drugs, we used data from a recent large-scale screen for drug synergy in melanoma. The authors screened for drug synergy using 108 × 108 drug combinations in the melanoma cell lines SK-MEL-28 and LOXIMVI^48^. By applying OptiCon to a melanoma gene expression dataset, we found significant overlap between our predicted synergistic gene pairs and target genes of synergistic drug pairs by the drug synergy study (hypergeometric test *p*-value = 1.4E–11, Supplementary Notes, Supplementary Data File 8). This result suggests that indeed OptiCon could identify targets of synergistic drugs although it does not use drug treatment data.

Certain genetic lesions are only observed in a subset of patients or subclones in the same tumor. Such inter- and intra-tumor genetic heterogeneities pose a significant challenge in cancer therapy^49^. As shown in Supplementary Fig. 6, the OCRs of synergistic OCNs are not only significantly enriched for known recurrently mutated cancer genes, but also have higher interaction densities between them than expected by chance. The higher interaction density increases the chance of pathway rewiring and development of drug resistance. Indeed, many genes in the OCRs that significantly contribute to the high interaction density are known to be involved in cancer therapy resistance (Fig. 7). Therefore, co-targeting synergistic OCNs could be an effective strategy to combat both disease heterogeneity and drug resistance.

Another interesting finding of our study is that experimentally derived cancer-type-specific and clinically relevant synthetic lethal interactions are enriched between OCRs of synergistic OCNs. Because synergistic OCNs can control both cancer genes and their synthetic lethal partners in the OCRs, co-targeting of the OCNs provides an effective way to combat both oncogene addiction and functional buffering due to synthetic lethal interactions. Moreover, because synthetic lethal interactions are enriched between OCRs of synergistic OCNs, genes in the OCRs are good candidates for a targeted screen for synthetic lethal interactions in a specific type of cancer.

Combination therapy is an effective therapeutic strategy not only for cancer, but also for other complex diseases. Because OptiCon only requires gene expression data, it is generally applicable to many complex diseases. For diseases not cancer, genes with disease-associated variants documented in the Online Mendelian Inheritance in Man (OMIM) database^50^ can be used for computing mutation scores in the identification of synergistic OCN pairs. Moreover, since OptiCon performs de novo prediction of targets for combination therapy, it is not limited to knowledge of existing drugs, which is far from comprehensive. Because the number of identified synergistic OCN pairs is small, they are suitable for targeted screen using RNAi and CRISPR/Cas9-based screening technologies^31,43^. Compared to genome-wide screen, such targeted screens are more cost-effective and versatile.

Besides gene expression data, other types of omics data should also be integrated in future development of OptiCon. For example, epigenetic information (e.g., DNA methylation and noncoding RNA expression) can be used to discover epigenetics-based^51^ combination therapy targets. It is also worth noting that the current limitation of all network-controllability-based methods is the inability of enumerating all SCCs for large networks. In this study, we used a set of 1000 SCCs for identifying direct control regions. Although most of the identified OCNs based on this set of SCCs are supported by multiple lines of evidence (HCC, 67%; LUAD, 61%; BRCA, 80%), more SCCs should be considered, if high-performance computing resources are available, in order to further improve the performance of OptiCon.

In summary, OptiCon represents a promising approach for identifying optimal and synergistic key regulators of deregulated pathways. Such regulators can serve as candidate targets for combination therapy to combat drug resistance and disease heterogeneity.

## Methods

### Construction of gene regulatory network

We constructed a human gene regulatory network by integrating annotations from three high-quality pathway databases: Reactome (1597 pathways)^24^, KEGG (195 pathways)^25^, and NCI-Nature Pathway Interaction Database (745 pathways)^26^. All pathways were downloaded in the Simple Interaction Format from Pathway Commons 2^52^. After removing undirected, redundant, and small-molecule-associated interactions, we obtained a regulatory network comprising 5959 nodes (genes) and 108,281 directed edges (regulatory links). The list of genes and their regulatory interactions is provided in Supplementary Data File 1.

### Gene expression datasets

RNA-Seq data used in this study were generated using tumor tissues and matched normal tissues from 50 HCC patients, 57 LUAD patients and 112 BRCA patients by The Cancer Genome Atlas consortium. For all three cancer types, read counts, fragments per kilobase of transcript per million mapped reads (FPKM) values and clinical information were downloaded from Genomic Data Commons (GDC, https://gdc-portal.nci.nih.gov/). Read counts and FPKM values were generated using GDC RNA-Seq pipeline (https://gdc.cancer.gov/about-data/data-harmonization-and-generation/genomic-data-harmonization/genomic-data-alignment/rna-seq-pipeline). We removed the batch effects on FPKM values using the *ComBat* function^53^ based on batch numbers extracted from the clinical information.

Gene expression data for melanoma was downloaded from Gene Expression Omnibus (GSE31909), which were generated using two melanoma cell lines SK-MEL-28 and LOXIMVI and two normal melanocyte lines, HEMn and HEMa. Each cell line has three replicates.

### Gene deregulation score (DScore)

Genes whose expression are significantly perturbed in diseased cells were identified using the edgeR algorithm^54^ and a false discovery rate cutoff ≤ 0.05. The DScores of these genes were defined as ${\mathrm{ - }}\log _{10}({\mathrm{adjusted}}\;{p} \hbox{-} {\mathrm{value}})$. The *p*-value was adjusted for multiple testing using the method of Benjamini–Hochberg^55^. For genes whose expression are not significantly perturbed, their DScores were set to zero.

### Control region of a gene

We define a control region for each gene to quantify the ability of the gene to control the dynamics of its downstream regulatory network. The control region can be divided into two parts, direct control region and indirect control region. For a gene *i* in the network, its direct control region includes itself and its downstream genes located in the SCC of the network. The downstream genes of gene *i* are genes to which there is a path from gene *i* in an SCC. There are multiple alternative SCCs for a given network^20^. For large networks (i.e., thousands of nodes), it is computationally intractable to examine all SCCs because enumerating all maximum matchings of a network is NP-hard. However, we noticed that the proportion of genes that have new direct control regions levels off when the number of different SCCs reaches 500 (Supplementary Fig. 1). The same trend was also observed for other real-world networks^20^. Therefore, the maximum matching algorithm was used here to obtain 1000 different SCCs for the regulatory network, which in turn were used to identify direct control regions of each gene (Supplementary Methods).

Because the directly controllable deregulated genes can influence their downstream genes, gene *i* can also have indirect control over additional genes. For identifying the indirect control region of gene *i*, we introduce an indirect control value (ICV) motivated by information flow theory^56^. For each deregulated gene *j* that is directly controlled by gene *i*, we first weight each edge <*u*, *v*> between genes *u* and *v* in the downstream subnetwork of gene *j* as below:

1 $$w_{ < u,v > }^j = \max ((|{\mathrm{corr}}({\mathrm{FPKM}}_j,{\mathrm{FPKM}}_u)| + |{\mathrm{corr}}({\mathrm{FPKM}}_j,{\mathrm{FPKM}}_v)|)/2,\;\varepsilon )$$

where $|{\mathrm{corr}}({\mathrm{FPKM}}_j,{\mathrm{FPKM}}_u)|$ and $|{\mathrm{corr}}({\mathrm{FPKM}}_j,{\mathrm{FPKM}}_v)|$ are the absolute values of the Pearson correlation coefficients between the FPKM values of genes *j* and *u* and of genes *j* and *v*. Following the study by Shih et al.^56^, we apply a minimum weight $\varepsilon$ since not all genes in the control region necessarily correlate with gene *j* in their expression. Given the sample size of each data set, we choose the value of $\varepsilon$ that corresponds to a *p*-value of 0.05 based on the theoretical distribution of the Pearson correlation coefficient. The sample sizes for the three cancer types are 100, 114, and 224 for liver, lung and breast cancer, respectively. The corresponding $\varepsilon$ values are 0.2, 0.18, and 0.13, respectively.

Given edge weights, the ICV of each gene (e.g., gene *k*) in the downstream subnetwork of gene *j*, denoted as ${\mathrm{ICV}}_k^j$, can be calculated as

2 $${\mathrm{ICV}}_k^j = 1/\mathop {\sum}\nolimits_{e \in {\mathrm{SP}}_{j \to k}} {( - \! \log w_e^j + 1)}$$

where $w_e^j$ denotes the weight of an edg*e e* in the downstream subnetwork of gene *j* computed as above and ${\mathrm{SP}}_{j \to k}$ represents the shortest path from gene *j* to gene *k*. If ${\mathrm{ICV}}_k^j$ is significantly high (above a given threshold *λ*), the genes in the shortest path from gene *j* to gene *k* are considered to be indirectly controlled by gene *i* due to the strong expression-correlated path from gene *j* to gene *k*. To determine the threshold *λ*, we generated a null distribution of ICVs using 100 randomized regulatory networks by randomly shuffling node labels. For all three cancer types, we used *λ* = 0.3 (empirical *p*-values < 0.01, Supplementary Fig. 2).

Given SCC and ICV, the control region of gene *i*, ${\mathrm{CR}}_i^{{\mathrm{SCC}}}$, can be formulated as following:

3 $$\begin{array}{l}{\mathrm{CR}}_i^{{\mathrm{SCC}}} = \overbrace {\{ i\} \cup \{ {\mathrm{Downstream}}\;{\mathrm{genes}}\;{\mathrm{of}}\;i\;{\mathrm{in}}\;{\mathrm{SCC}}\} }^{{\mathrm{Direct}}\;{\mathrm{control}}\;{\mathrm{region}}} \cup \\ \quad \quad \quad \,\,\,\underbrace {\{ {\mathrm{genes}}\;{\mathrm{in}}\;{\mathrm{the}}\;{\mathrm{shortest}}\;{\mathrm{path}}\;{\mathrm{from}}\;j\;{\mathrm{to}}\;k,\;{\mathrm{if}}\;{\mathrm{ICV}}_k^j \ge \lambda \} }_{{\mathrm{Indirect}}\;{\mathrm{control}}\;{\mathrm{region}}}\end{array}$$

where *j* is a deregulated gene that is directly controlled by gene *i* and *k* is a gene located in the downstream subnetwork of gene *j*. Note that genes in a control region are unique while direct and indirect control regions may overlap. Here, we only consider indirect control regions of deregulated genes because we attempted to identify an OCR (see below) that maximizes the deregulation in both direct and indirect control regions. If indirect control regions are also considered for non-deregulated genes, we might obtain an OCR with large amount of deregulation in the indirect control part but small amount of deregulation in the direct control part.

### A greedy search algorithm to identify OCNs

Since control regions of different genes may overlap, we formulate the identification of OCNs and their OCRs as a combinatorial optimization problem. The objective function consists of three variables: *o*, *d* and *u*, representing the optimal influence, desired influence and undesired influence by the candidate OCNs, respectively. The desired influence *d* is defined as the fraction of deregulation that can be controlled by OCNs, while the undesired influence *u* is defined as the fraction of controllable genes that are not deregulated in disease.

4 $$d = \frac{{\mathop {\sum}\limits_{i \in {\mathrm{OCRs}}} {{\mathrm{DScore}}_i} }}{{\mathop {\sum}\limits_{i \in G} {{\mathrm{DScore}}_i} }}$$

5 $$u = \frac{{|\{ i|{\mathrm{DScore}}_i = 0,\;i \in {\mathrm{OCRs}}\} |}}{{|\{ i|{\mathrm{DScore}}_i = 0,\;i \in G\} |}}$$

where *G* represents a gene regulatory network. The objective is to identify a set of OCNs that maximizes the optimal influence $o = d - u$.

We employ a greedy search algorithm to solve this optimization problem. The pseudo code for the algorithm is provided in Supplementary Methods. For each candidate gene, the algorithm accepts it as an OCN if its addition leads to an improved optimal influence value. We calculate the growth rate of optimal influence as $\left( {\frac{{o_{{\mathrm{new}}} \, - \, o_{{\mathrm{previous}}}}}{{o_{{\mathrm{previous}}}}}} \right)$ for each candidate OCN. We found that the growth rate does not change significantly once it drops below 5% (Supplementary Fig. 3). We thus used 5% as the stopping criterion of the greedy search. To determine if the result of a greedy search is a suboptimal solution, we initiate multiple searches from the control nodes of the top 0.01% of all control regions in a network (see Supplementary Methods for details, Supplementary Fig. 3a, c, e).

### False discovery rate of OCNs

We compute the false discovery rate of identified OCNs based on OCN occurrence frequencies in real and randomized regulatory networks (Supplementary Fig. 3b, d, f). Occurrence frequency of an OCN is computed as the number of greedy search solutions containing this OCN divided by the total number of greedy searches. The null distribution of OCN occurrence frequency was generated using 10 randomized regulatory networks.

### Identifying synergistic OCN pairs

We introduce a metric, synergy score, to measure the synergistic interaction between two OCNs. Motivated by our current understanding of the mechanisms of synergistic drug combinations^57^ and acquired drug resistance^1^, the synergy score between two OCNs (e.g., ${\mathrm{OCN}}_p$ and ${\mathrm{OCN}}_q$) consists of two parts. The mutation score measures the enrichment of recurrently mutated cancer genes in the OCR of each OCN. The crosstalk score measures the interaction density between genes in the two OCRs.

6 $${\mathrm{Synergy}}\;{\mathrm{score}} = {\mathrm{Norm}}({\mathrm{Mutation}}\;{\mathrm{score}}) \times {\mathrm{Norm}}({\mathrm{Crosstalk}}\;{\mathrm{score}})$$

7 $$	{\mathrm{Mutation}}\;{\mathrm{score}} \\ 	= \sqrt {( - \! \log \;{\mathrm{Enrichment}}\;{p} \hbox{-} {\mathrm{value}}_{{\mathrm{OCR}}_p}) \times ( - \! \log \;{\mathrm{Enrichment}}\;{p} \hbox{-} {\mathrm{value}}_{{\mathrm{OCR}}_q})}$$

8 $${\mathrm{Crosstalk}}\;{\mathrm{score}} = {\mathrm{Interaction}}\;{\mathrm{density}}({\mathrm{OCR}}_p,{\mathrm{OCR}}_q)$$

Annotation for cancer (driver) genes were downloaded from the Cancer Gene Census database^58^, which were confirmed to have recurrent somatic mutations in the specific cancer type using data from the Catalogue of Somatic Mutations in Cancer (COSMIC) database^47^ (Supplementary Data File 2). Interaction density between ${\mathrm{OCR}}_p$ and ${\mathrm{OCR}}_q$ is defined as the ratio of the number of observed links over all possible directed links between the nodes in ${\mathrm{OCR}}_p$ and ${\mathrm{OCR}}_q$. The synergy score equals the product of the min-max normalized mutation and crosstalk scores. To identify significantly synergistic OCN pairs, we generated a null distribution of synergy scores based on 10 million randomly selected gene pairs from the input regulatory network. The OCN pairs with an empirical *p*-value < 0.05 were predicted as significantly synergistic. *p*-values were adjusted for multiple testing using the method of Benjamini–Hochberg^55^.

### Performance comparison with TargetControl, VIPER, and RACS

TargetControl^16^ is a network controllability-based method for identifying a minimal set of control nodes that can efficiently control a pre-selected set of nodes in a network. However, it only predicts a minimal set of control nodes in a network, instead of synergistic gene pairs. We included TargetControl as a baseline comparison. To run TargetControl, we selected a subset of top deregulated genes based on their deregulation scores as the pre-selected node set, which has the same size as the deregulated gene set in OCRs identified by OptiCon. Since TargetControl requires a directed acyclic network as the input, we generated 10 acyclic regulatory networks by removing 10 different minimum feedback edge sets from our constructed regulatory network. Because multiple maximum matchings of a network exist, 100 different control node sets were identified in each acyclic regulatory network. Taken together, for each cancer type, 1000 control node sets were identified by TargetControl and compared with OCNs identified by OptiCon.

VIPER^12^ uses the Master Regulator Inference algorithm^13^ to identify synergistic master regulators based on gene expression data and a gene regulatory network. For each cancer type, we used the same gene expression data and network as inputs to VIPER. Master regulators were first identified using the *msviper* function and adjusted *p*-values < 0.05. Identified master regulators were used as input to the *msviperCombinatorial* and *msviperSynergy* functions for synergy analysis. Synergistic master regulators with adjusted *p*-values < 0.05 and their target subnetworks were identified for comparison.

RACS^9^ is a semi-supervised learning method that combines drug pharmacological characteristics, drug-targeted networks and transcriptomic profiles to identify potential synergistic combinations of existing cancer drugs. The synergistic gene pairs for comparison were defined as the pair-wise target combinations of RACS-predicted synergistic drugs. Since RACS does not identify a target subnetwork like OptiCon and VIPER, we used the OCR for each target of RACS-predicted drugs as the target subnetwork.

### Enrichment of synthetic lethal interactions between OCRs

Experimentally derived cancer-type-specific synthetic lethal interactions were downloaded from the SynLethDB database^30^ and a recent study using CRISPR-Cas9 screen^31^ (Supplementary Data File 3). Clinically relevant synthetic lethal interactions were downloaded from a recent study^32^. The enrichment p-value of synthetic lethal interactions between the OCRs of an OCN pair was computed using the hypergeometric distribution based on the following four numbers: (1) the number of synthetic lethal interactions between two OCRs; (2) the total number of gene pairs between two OCRs; (3) the number of all synthetic lethal interactions in the input regulatory network; (4) the total number of gene pairs in the network. *P*-values were adjusted for multiple testing using the method of Benjamini–Hochberg^55^.

### Crosstalk genes affecting interaction density between OCRs

Crosstalk genes are defined as those that are incident on interactions between OCRs of two OCNs. The effect of a crosstalk gene on the interaction density between two OCRs (Δ*D*) is quantified as the decrease in interaction density of two OCRs after the crosstalk gene is removed from the OCR. Empirical *p*-value of Δ*D* is calculated using a null distribution of Δ*D *of crosstalk genes controlled by one million randomly selected gene pairs from the input regulatory network.

### Cell lines and cloning of CRISPR-based knockout constructs

SkHep1 and A549 cell lines stably expressing Cas9 endonuclease (SkHep1-Cas9^+^ and A549-Cas9^+^) are gifts from Junwei Shi (University of Pennsylvania). MCF7 cell line stably expressing Cas9 (MCF7-Cas9^+^) was purchased from Applied Biological Materials (Cat # T3257). Both SkHep1-Cas9^+^ and A549-Cas9^+^ cells were maintained in DMEM media and MCF7-Cas9^+^ cells were maintained in PriGrowIII media (TM003, abm). All cell culture media was supplied with 10% FBS, 100 unit/ml penicillin and 100 μg/ml streptomycin.

sgRNAs targeting test genes were designed using the DESKGEN software (https://www.deskgen.com/landing/), and sequences for non-targeting control sgRNAs were chosen from published literature^59^. The sgRNAs targeting test genes or control sgRNAs were individually cloned into a lentiviral vector, either pMCB306 (Addgene: #89360) which expresses the GFP reporter gene or pMCB320 (Addgene: #89359) which expresses the mCherry reporter gene. All cloned sgRNA constructs were verified by Sanger sequencing.

### Lentivirus production and transduction

Lentiviruses were produced by transfecting HEK293FT cells with the packaging plasmids pMD2.G and psPAX2, and individual sgRNA constructs. Lentivirus-laden supernatant was harvested 48 h after transfection. Viral supernatant was filtered through 0.45 μm polyvinylidene difluoride filter (Millipore), aliquoted and frozen at −80^ °^C.

### CRISPR-based double knockout growth assay

To make double and single knockouts, we used the sgRNA-based double infection protocol^43^ with minor modifications. The Cas9-expressing cells were double infected with pooled viral supernatant (pMCB306 + pMCB320 vectors carrying individual sgRNAs) with 8 μg/ml polybrene (Millipore) by spin-infection. sgRNA sequences are listed in Supplementary Data File 7.

Four days after viral transduction, cells were detached with 0.25% Trypsin-EDTA (ThermoFisher) and seeded into three replicate wells, each with equal number of cells. For each replicate, the fraction of each group of cells (GFP^+^, mCherry^+^, GFP^+^ mChery^+^, and GFP^−^ mCherry^−^) was counted by FACS and designated as the *T*_b_ data. At the end of culturing for another 5–10 days, fraction of each group of cells was counted again by FACS and designated as the *T*_e_ data.

The following equation was used to calculate the growth phenotypes of both single or double knockout populations:

9 $$\log _2\left(\frac{{f_{{\mathrm{ko}}}^{\mathrm{e}}/f_{{\mathrm{wt}}}^{\mathrm{e}}}}{{f_{{\mathrm{ko}}}^{\mathrm{b}}/f_{{\mathrm{wt}}}^{\mathrm{b}}}}\right)/d$$

where $f_{{\mathrm{wt}}}^{\mathrm{b}}$ is the fraction of wild-type cells at the beginning of the assay; $f_{{\mathrm{ko}}}^{\mathrm{b}}$ is the fraction of knockout cells (either single or double knockout) at the beginning of the assay; $f_{{\mathrm{wt}}}^{\mathrm{e}}$ is the fraction of wild-type cells at the end of the assay; $f_{{\mathrm{ko}}}^{\mathrm{e}}$ is the fraction of knockout cells at the end of the assay; *d* is the number of doublings of either the single or the double knockout cells.

The number of doublings is calculated using the following formula,

10 $$d_{{\mathrm{ko}}} = d_{{\mathrm{wt}}}/\left(1 - \frac{x}{T}d_{{\mathrm{wt}}}\right)$$

where $d_{{\mathrm{ko}}}$ and *d*_wt_ are the doubling times of knockout and wild-type cells, respectively; *T* is the total amount of time of the assay; and *x* is a normalizing factor. *x* is calculated as following: $x = \log _2(f_{{\mathrm{ko}}}^{\mathrm{b}} \cdot f_{{\mathrm{wt}}}^{\mathrm{e}}/f_{{\mathrm{wt}}}^{\mathrm{b}} \cdot f_{{\mathrm{ko}}}^{\mathrm{e}})$. The doubling time of SkHep1 cells (30 h), A549 cells (22 h), and MCF7 cells (50 h) were obtained from either ATCC or DSMZ.

Genetic interaction (GI) score was calculated as the difference between the observed growth phenotype of the double positive population (the GFP^+^mCherry^+^ population) and the expected growth phenotype of the double positive population (the sum of GFP^+^ only and mCherry^+^ only populations). Finally, to determine if significant synergy exists between a gene pair, the GI score of gene-gene double knockout population was compared to the safe-gene single knockout populations, using one-sided Student’s *t*-test.

### Degree-based method for identifying control nodes

We define a regulatory value for each node in the input network as the difference between the out-degree and the in-degree of the node. For identifying the regulatory nodes with significantly large regulatory values, we construct a null distribution of regulatory values using 100 randomized networks. The nodes that can change the number of regulatory nodes in a network play an important role in controlling the degree distribution of the network and thus can be considered as degree-based control nodes. Based on this assumption, we remove each node in the network and re-compute the set of significant regulatory nodes. The set of control nodes is identified as those whose elimination from the network increases the number of regulatory nodes in the network.

### Survival analysis of OCN pairs

We downloaded RNA-Seq and clinical information of HCC patients, LUAD patients and BRCA patients from The Cancer Genome Atlas data portal (TCGA). For each OCN pair, we used the *coxph* function from the *survival* R package to fit a Cox proportional hazards model^60^ with the time to death as the event. Seven covariates were considered in the Cox model, including gene expression levels of two OCNs, an interaction term for the two OCNs, patient age, tumor subtype, tumor stage and gender. The *p*-value of the regression coefficient for the interaction term was used to evaluate clinical relevance of an OCN pair to patient survival.

### Reporting summary

Further information on research design is available in the Nature Research Reporting Summary linked to this article.

## Supplementary information

Supplementary Information

Reporting Summary

Description of Additional Supplementary Files

Supplementary Data 1

Supplementary Data 2

Supplementary Data 3

Supplementary Data 4

Supplementary Data 5

Supplementary Data 6

Supplementary Data 7

Supplementary Data 8

Supplementary Data 9

Source Data

## Data Availability

The authors declare that the main data supporting the findings of this study are available within the article and its Supplementary Information. All other relevant data are available upon request.

## Code availability

A software package implementing the OptiCon algorithm has been deposited at GitHub (https://github.com/tanlabcode/OptiCon).
