# Supplementary material for: Optimal control nodes in disease-perturbed networks as targets for combination therapy
Source: Nat Commun. 2019 May 16;10:2180. doi: 10.1038/s41467-019-10215-y (PMC6522545; doi:10.1038/s41467-019-10215-y)
Supplement: Supplementary file 1 — Supplementary Information [file 41467_2019_10215_MOESM1_ESM.pdf]

Supplementary Information

Optimal control nodes in disease-perturbed networks as targets for combination therapy

Hu et al.

Supplementary Figures

Supplementary Figure 1. Proportion of genes that have new direct control regions as a function of the number of structural control configurations (SCCs) of the network.
A human gene regulatory network with 5,959 genes and 108,281 regulatory interactions was used as the input for searching SCCs and direct control regions of all genes. The figure shows that the proportion of genes that have new direct control regions starts to level off when the number of SCCs reaches 500. Red line, fitted curve for the scatter plot (grey dots).

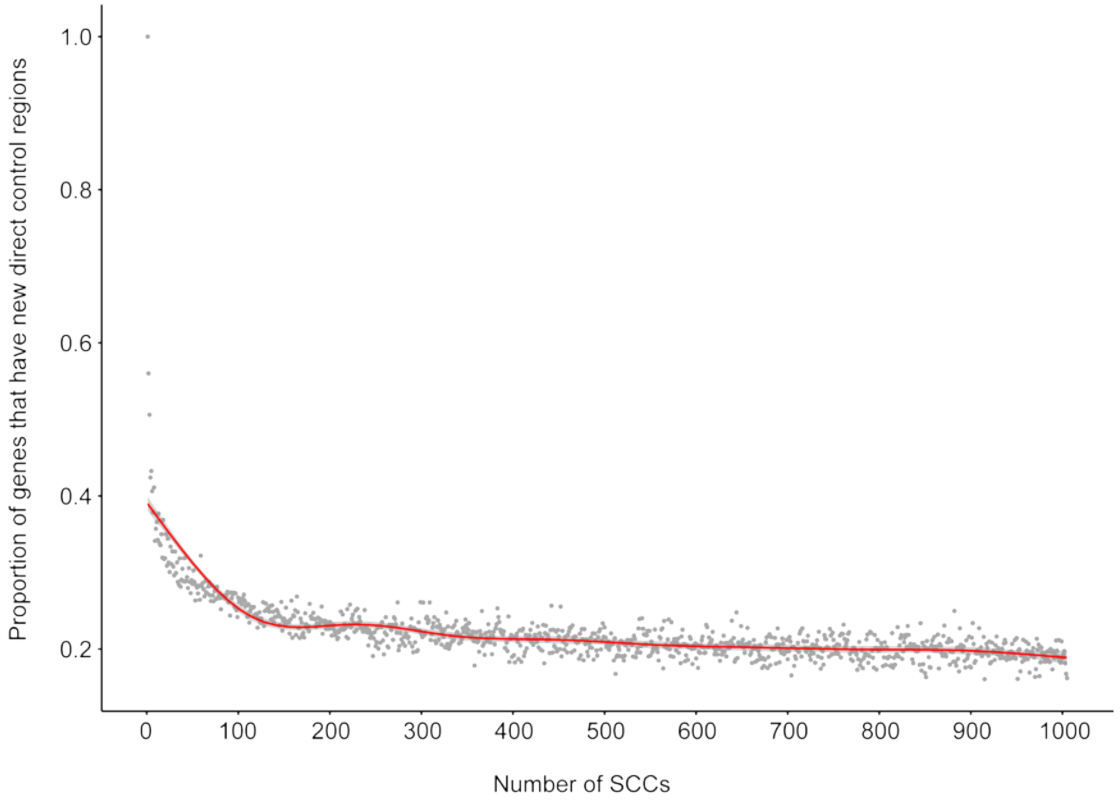

Supplementary Figure 2. Determination of the threshold for the indirect control value.

Indirect control region of a gene is identified based on indirect control values of its downstream genes. The indirect control value ranges from 0 to 1. Dashed line, value of the threshold (λ) that corresponds to an empirical p-value < 0.01; Solid line, null distribution of indirect control values generated using 100 randomized regulatory networks. **(a)** Liver cancer. **(b)** Lung cancer. **(c)** Breast cancer.

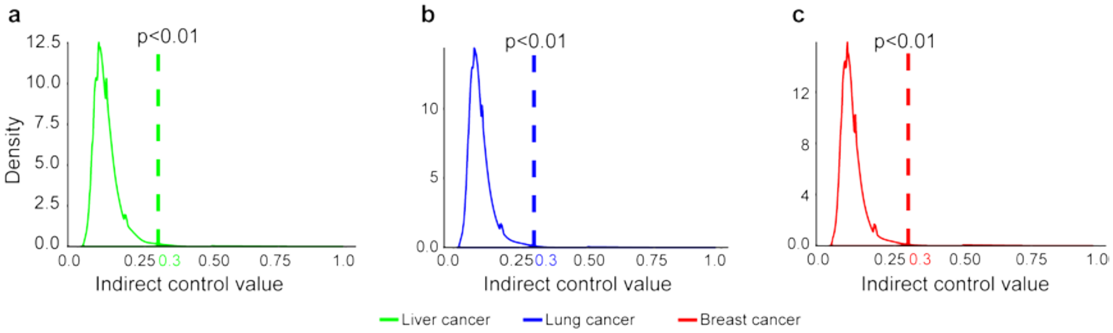

Supplementary Figure 3. Procedures used to determine parameters of the greedy search algorithm for finding a set of optimal control nodes (OCNs).
(a, c, e) Determination of the stopping criterion based on the growth rate of the optimal influence value. To initiate multiple greedy searches, all control regions of nodes in a regulatory network are ranked based on their optimal influence value (i.e. desired influence minus undesired influence). Each control node of the top 0.01% of all control regions is used as the starting point for a greedy search. HCC, hepatocellular carcinoma; LUAD, lung adenocarcinoma; BRCA, breast invasive carcinoma. In total, 532, 595, and 595 greedy searches are performed for HCC, LUAD, and BRCA network, respectively. Each colored line represents the growth rate of the optimal influence value during a greedy search initiated from a control node. As shown in the figure, the growth rate levels off at 5% for all three regulatory networks. Thus, 5% is chosen as the stopping criterion for the greedy search. **(b, d, f)** Calculation of false discovery rate of OCNs based on OCN occurrence frequencies in real and randomized regulatory networks. Shown are cumulative distribution function (CDF) of OCN occurrence frequency in each regulatory network. Occurrence frequency of an OCN is computed as the number of greedy search solutions containing this OCN divided by the total number of greedy searches. Red line, CDF of OCN occurrence frequency generated using the real regulatory network. Orange line, CDF of OCN occurrence frequency generated using 10 randomized regulatory networks. Blue dashed line, false discovery rate of 0.05 for predicting OCNs.

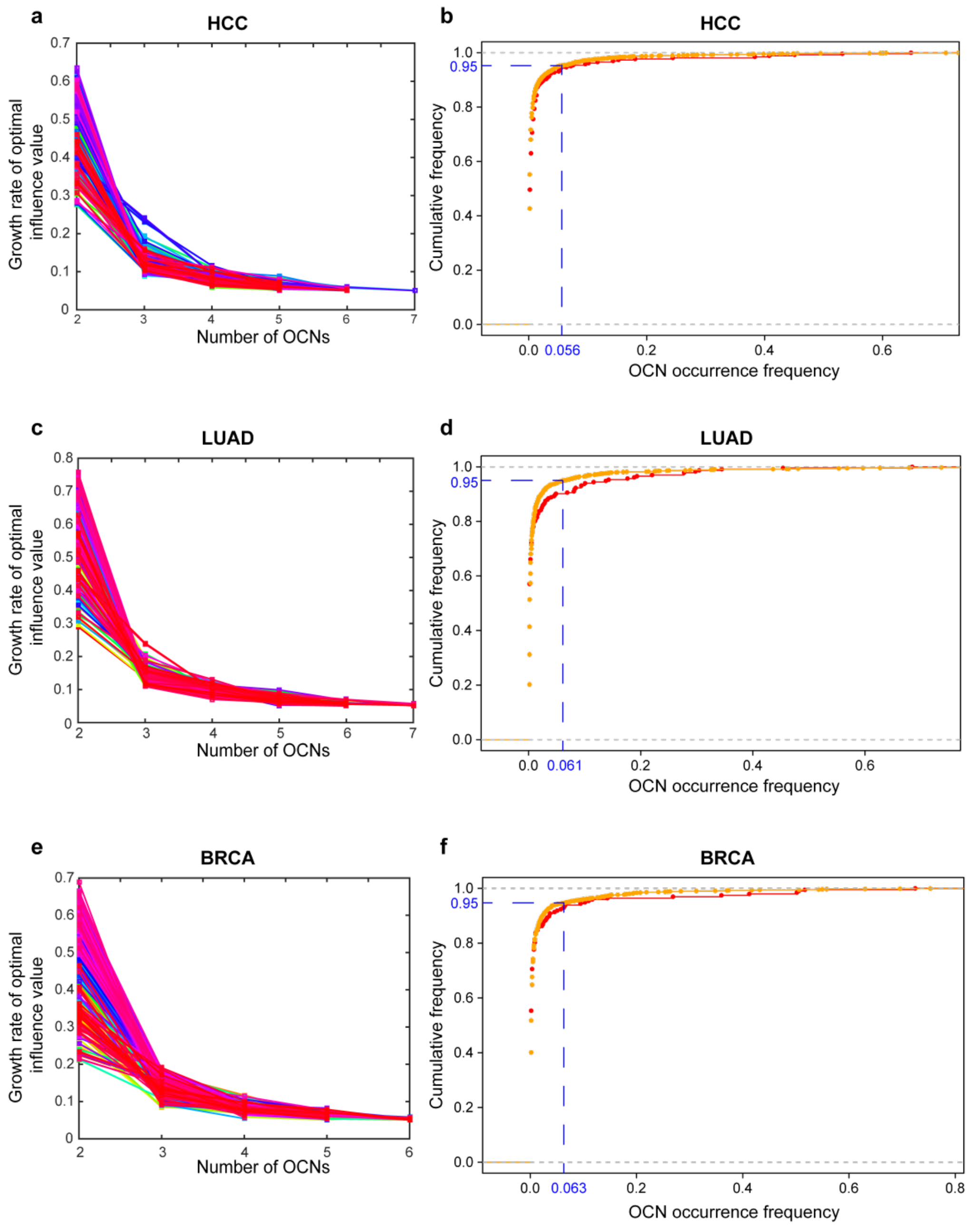

a
HCC
Growth rate of optimal influence value
Number of OCNs
b
HCC
Cumulative frequency
0.95
0.056
OCN occurrence frequency
c
LUAD
Growth rate of optimal influence value
Number of OCNs
d
LUAD
Cumulative frequency
0.95
0.061
OCN occurrence frequency
e
BRCA
Growth rate of optimal influence value
Number of OCNs
f
BRCA
Cumulative frequency
0.95
0.063
OCN occurrence frequency

Supplementary Figure 4. Clinically relevant synthetic lethal interactions are enriched between optimal control regions of synergistic OCN pairs.

(a, c, e) Clinically relevant synthetic lethal interactions (cSL) are significantly enriched between optimal control regions (OCRs) of 52 (68%), 89 (46%) and 49 (78%) synergistic OCN pairs identified in hepatocellular carcinoma (HCC), lung adenocarcinoma (LUAD) and breast invasive carcinoma (BRCA), respectively (hypergeometric test p-values < 0.05). Shade of magenta in the heatmap is inversely proportional to the enrichment p-values. **(b, d, f)** Contingency tables and corresponding Fisher's exact test p-values are shown for HCC, LUAD and BRCA, respectively, indicating that clinically relevant synthetic lethal interactions are more enriched between OCRs of synergistic OCN pairs than non-synergistic OCN pairs.

a HCC

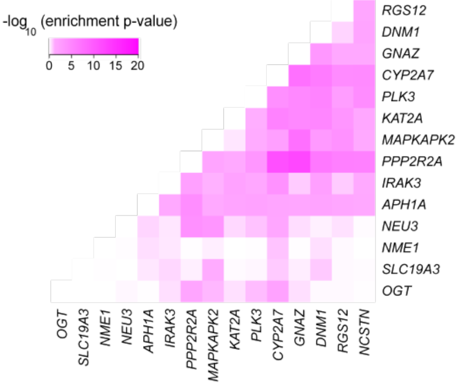

b HCC

	Synergistic	Non-synergistic
cSL-enriched	52 pairs	1 pair
not cSL-enriched	25 pairs	27 pairs

Fisher exact test p = 1.1E-9

c LUAD

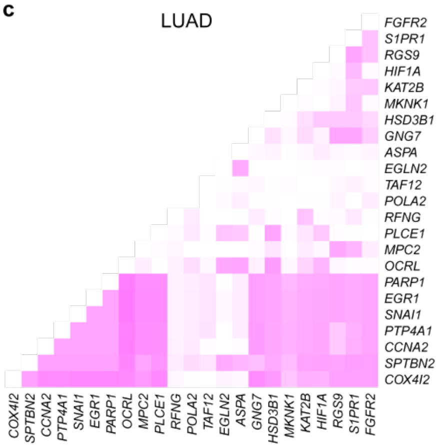

d LUAD

	Synergistic	Non-synergistic
cSL-enriched	89 pairs	4 pairs
not cSL-enriched	103 pairs	57 pairs

Fisher exact test p = 2.0E-9

e BRCA

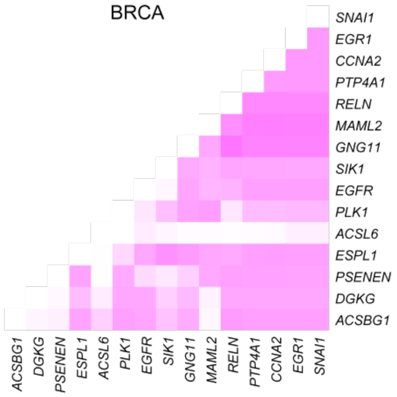

f BRCA

	Synergistic	Non-synergistic
cSL-enriched	49 pairs	17 pairs
not cSL-enriched	14 pairs	25 pairs

Fisher exact test p = 1.7E-4

Supplementary Figure 5. Representative FACS plot of cells infected with lentiviruses expressing sgRNAs targeting gene pairs in liver cancer (a), lung cancer (b) and breast cancer (c).

Value in each quadrant indicates the percentage of cells expressing a given reporter in the culture. The growth phenotype is calculated by measuring the relative depletion of the single-infected and double-infected cells between the start and the end of the growth assay. KO, single knockout. DKO, double knockout. RFU, relative fluorescence unit. Source data are provided as a Source Data file.

a

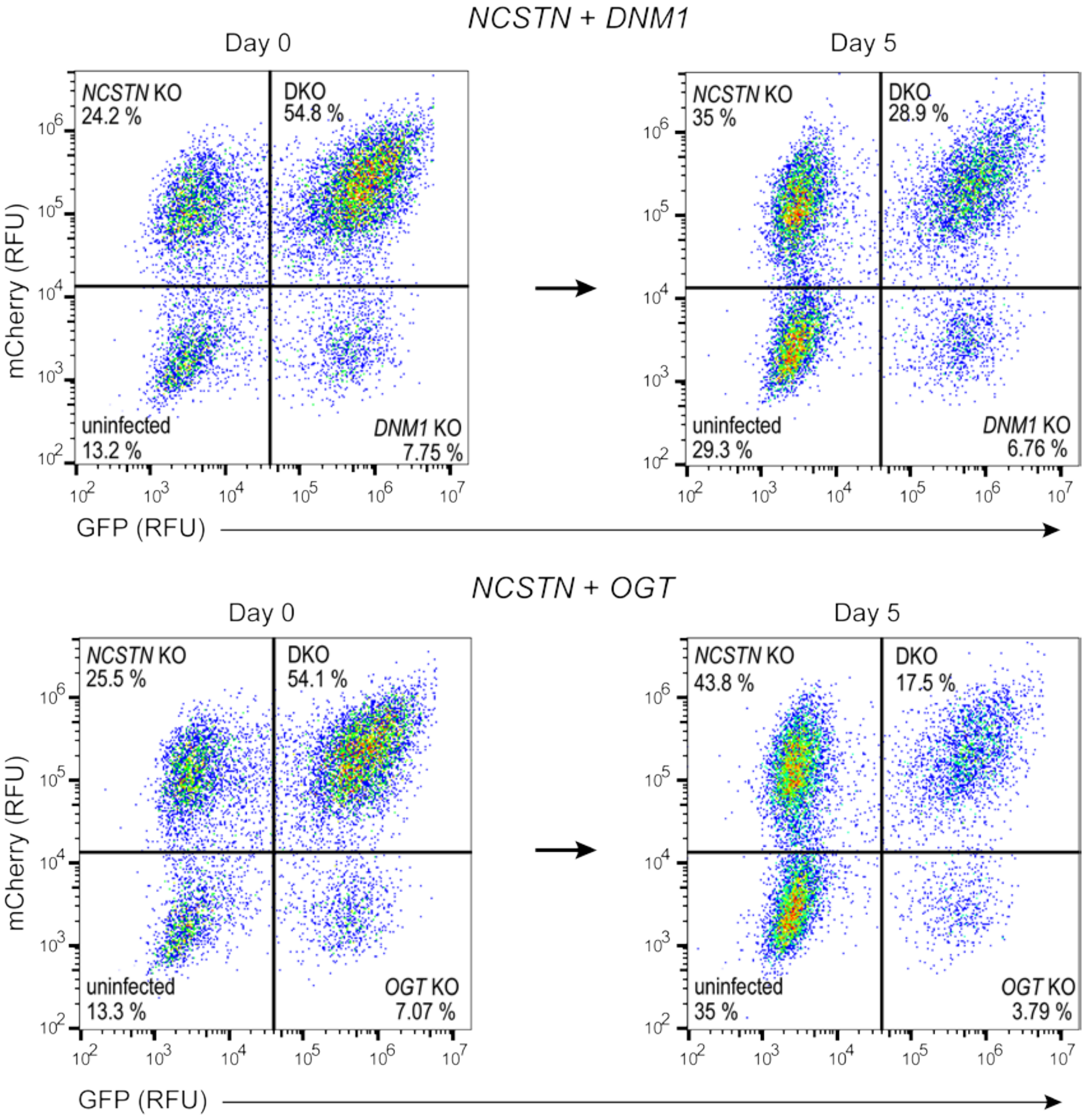

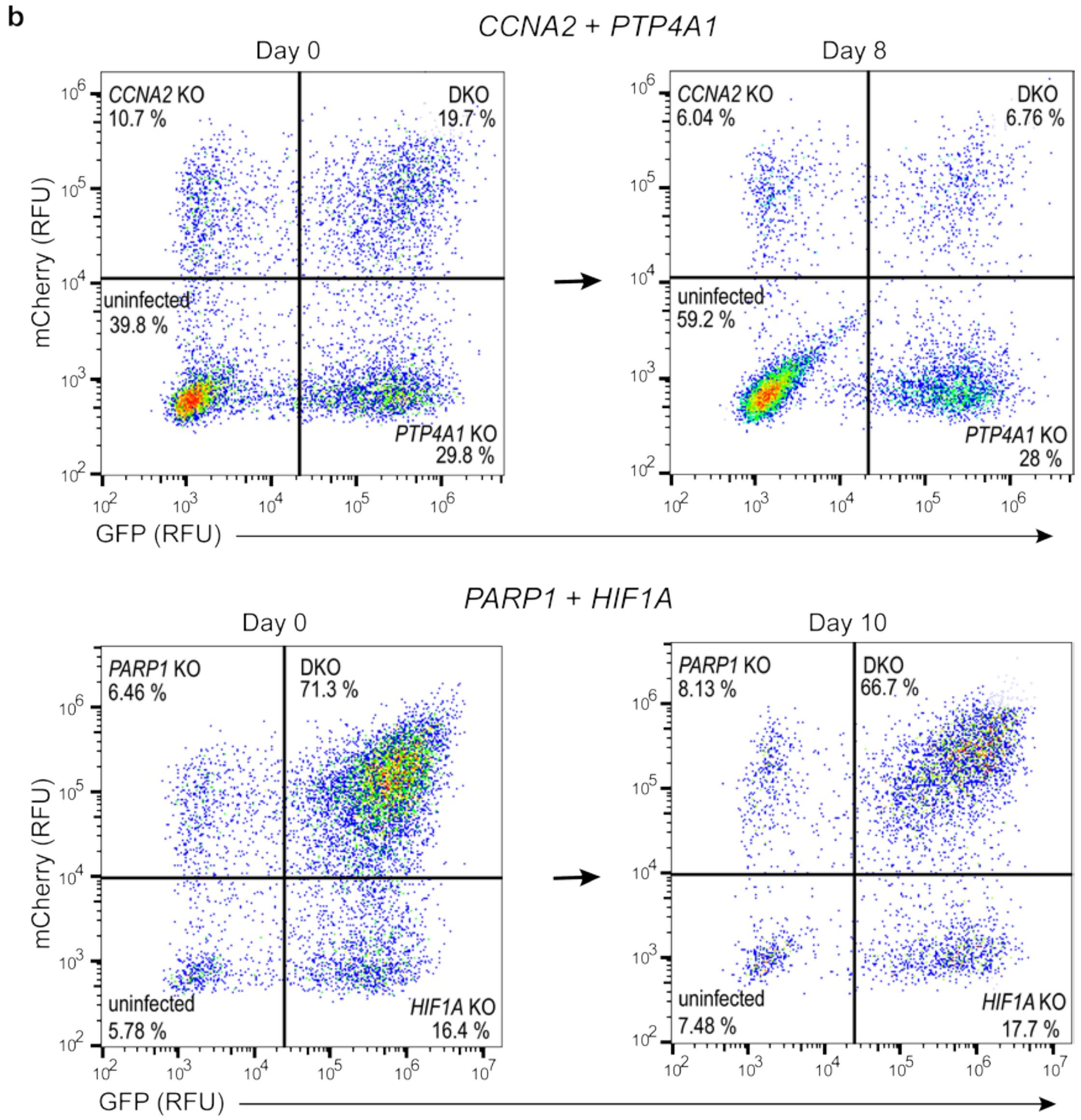
b
CCNA2 + PTP4A1
Day 0
Day 8
CCNA2 KO
10.7 %
DKO
19.7 %
uninfected
39.8 %
PTP4A1 KO
29.8 %
CCNA2 KO
6.04 %
DKO
6.76 %
uninfected
59.2 %
PTP4A1 KO
28 %
mCherry (RFU)
GFP (RFU)
PARP1 + HIF1A
Day 0
Day 10
PARP1 KO
6.46 %
DKO
71.3 %
uninfected
5.78 %
HIF1A KO
16.4 %
PARP1 KO
8.13 %
DKO
66.7 %
uninfected
7.48 %
HIF1A KO
17.7 %
mCherry (RFU)
GFP (RFU)

c

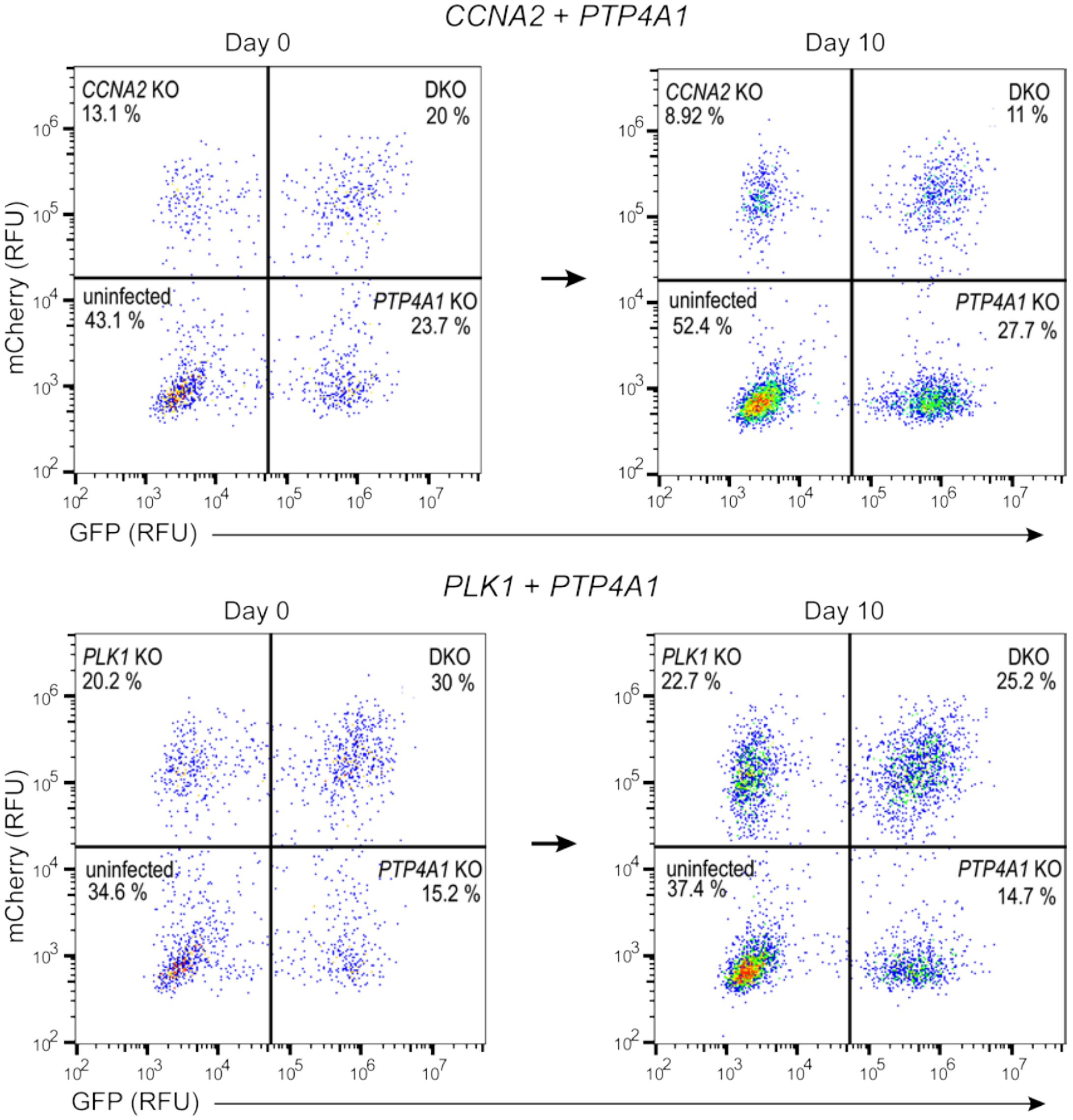

CCNA2 + PTP4A1
Day 0
Day 10
CCNA2 KO
13.1 %
DKO
20 %
uninfected
43.1 %
PTP4A1 KO
23.7 %
CCNA2 KO
8.92 %
DKO
11 %
uninfected
52.4 %
PTP4A1 KO
27.7 %
mCherry (RFU)
GFP (RFU)
PLK1 + PTP4A1
Day 0
Day 10
PLK1 KO
20.2 %
DKO
30 %
uninfected
34.6 %
PTP4A1 KO
15.2 %
PLK1 KO
22.7 %
DKO
25.2 %
uninfected
37.4 %
PTP4A1 KO
14.7 %
mCherry (RFU)
GFP (RFU)

Supplementary Figure 6. Optimal control regions controlled by synergistic OCN pairs are enriched for recurrently mutated cancer genes and have higher interaction densities than expected by chance.

(a, b, c) Recurrently mutated cancer genes in a specific cancer type are significantly enriched (hypergeometric test p-values < 0.05) in optimal control regions (OCRs) controlled by two synergistic OCNs. In total, OCRs of 77 (100%), 189 (98%) and 63 (100%) synergistic OCN pairs are enriched for recurrently mutated cancer genes in hepatocellular carcinoma (HCC), lung adenocarcinoma (LUAD) and breast invasive carcinoma (BRCA), respectively. Shade of yellow in the heatmap is inversely proportional to the enrichment p-values. **(d, e, f)** Dense interactions among OCRs of synergistic OCN pairs (empirical p-values < 0.1). In total, OCRs of 61 (79%), 160 (83%) and 56 (89%) synergistic OCN pairs have significantly higher interaction density in HCC, LUAD and BRCA, respectively. Empirical p-value for interaction density is calculated using a null distribution generated based on 10 million randomly selected gene pairs from the input regulatory network. Shade of green in the heatmap is inversely proportional to the empirical p-values.

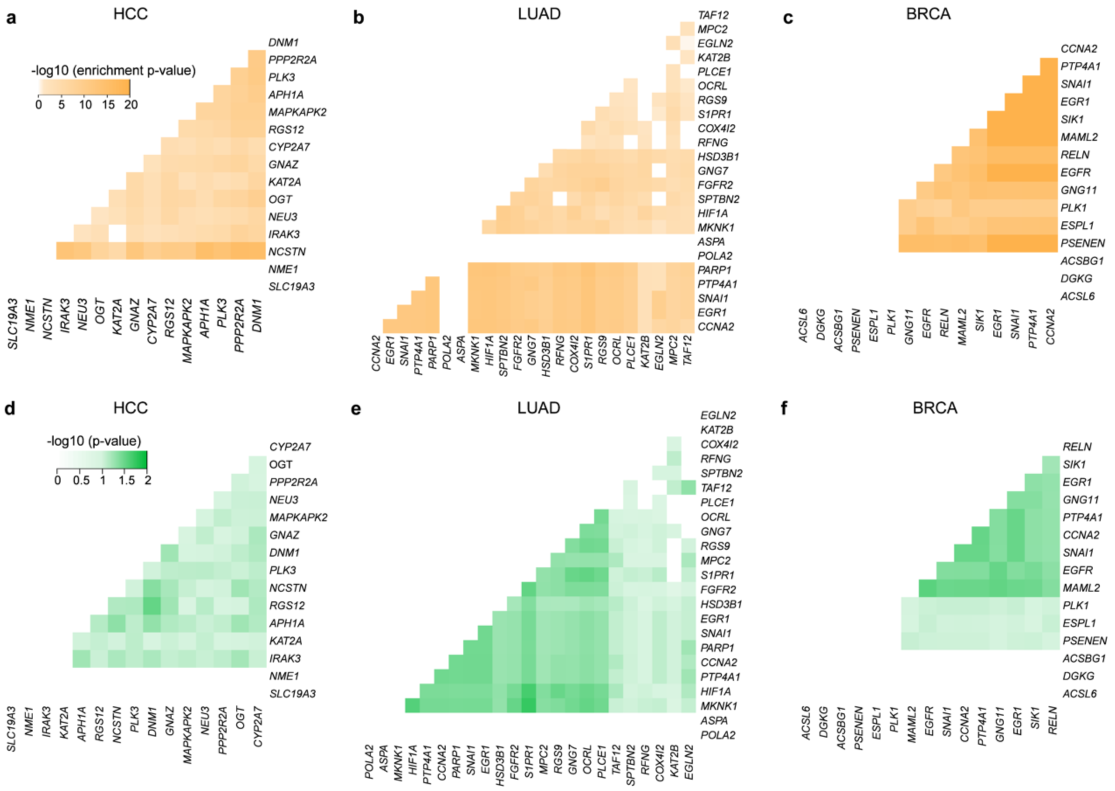

a
HCC
-log10 (enrichment p-value)
0 5 10 15 20
DNM1
PPP2R2A
PLK3
APH1A
MAPKAPK2
RGS12
CYP2A7
GNAZ
KAT2A
OGT
NEU3
IRAK3
NCSTN
NME1
SLC19A3
b
LUAD
TAF12
MPC2
EGLN2
KAT2B
PLCE1
OCRL
RGS9
S1PR1
COX4I2
RFNG
HSD3B1
GNG7
FGFR2
SPTBN2
HIF1A
MKNK1
ASPA
POLA2
PARP1
PTP4A1
SNAI1
EGR1
CCNA2
c
BRCA
CCNA2
PTP4A1
SNAI1
EGR1
SIK1
MAML2
RELN
EGFR
GNG11
PLK1
ESPL1
PSENEN
ACSBG1
DGKG
ACSL6
d
HCC
-log10 (p-value)
0 0.5 1 1.5 2
CYP2A7
OGT
PPP2R2A
NEU3
MAPKAPK2
GNAZ
DNM1
PLK3
NCSTN
RGS12
APH1A
KAT2A
IRAK3
NME1
SLC19A3
e
LUAD
EGLN2
KAT2B
COX4I2
RFNG
SPTBN2
TAF12
PLCE1
OCRL
GNG7
RGS9
MPC2
S1PR1
FGFR2
HSD3B1
EGR1
SNAI1
PARP1
CCNA2
PTP4A1
HIF1A
MKNK1
ASPA
POLA2
f
BRCA
RELN
SIK1
EGR1
GNG11
PTP4A1
CCNA2
SNAI1
EGFR
MAML2
PLK1
ESPL1
PSENEN
ACSBG1
DGKG
ACSL6

Supplementary Methods

Identification of a maximum matching of a directed network

For identifying a maximum matching of a directed network *G* (Fig. 1a, left panel), we first converted the network *G* to a bipartite graph (Fig. 1a, middle panel). Each node in the network is represented by two types of nodes: a start node (+) and an end node (-). The directed edges can only connect from start nodes to end nodes according to the relationship in the original network.

The identification of a maximum matching of the network *G* is equivalent to finding a maximum matching of its associated bipartite graph (Fig. 1a, middle panel), which can be solved using the Hungarian algorithm [1]. A matching of a bipartite graph is a subset of the edges in the graph, in which any two edges do not share common starting nodes or ending nodes. A matching of the maximum size is called a maximum matching. Note that the maximum matching of a network is not unique, but its size is uniquely determined by the network topology.

Pseudo code of the greedy search algorithm for identifying optimal control nodes (OCNs) and their optimal control regions (OCRs)

Inputs: A DScore-weighted gene regulatory network *G*, a threshold α for the growth rate of optimal influence value;

Outputs: OCNs & OCRs

---

1. Identify *M* different structural control configurations $\mathrm{SCC}_m\ (m=1,2,\ldots,M)$ for

the network G using the maximum matching algorithm [1];

2. For each SCC, identify the control region (CR) of each gene. Here, we assume that there are a total of N genes in the network G.
3. Rank all $N \times M$ control regions identified in Step 2 based on their optimal influence values (i.e. desired influence minus undesired influence) and use each control node p of the top 0.01% of all control regions as the starting point for a search below (i.e. initiate $\mathrm{OCNs} = \{p\}$, $\mathrm{OCRs} = \mathrm{CR}_p$);
4. Search from the remaining control regions to identify the $\mathrm{CR}_q^{\mathrm{SCC}_m}$ (the control region of node q in SCC_m) that maximizes the optimal influence value $o_{\mathrm{OCRs} \cup \mathrm{CR}_q^{\mathrm{SCC}_m}}$;
5. If the growth rate of optimal influence value no less than α (i.e. $\frac{o_{\mathrm{OCRs} \cup \mathrm{CR}_q^{\mathrm{SCC}_m}} - o_{\mathrm{OCRs}}}{o_{\mathrm{OCRs}}} \geq \alpha$), then update $\mathrm{OCNs} = \mathrm{OCNs} \cup \{q\}$, $\mathrm{OCRs} = \mathrm{OCRs} \cup \mathrm{CR}_q^{\mathrm{SCC}_m}$, $o_{\mathrm{OCRs}} = o_{\mathrm{OCRs} \cup \mathrm{CR}_q^{\mathrm{SCC}_m}}$, and go Step 4. Otherwise, terminate the algorithm and return OCNs and OCRs.

It is worth noting that we used a set of 1,000 structural control configurations (SCCs) for identifying direct control regions in this study since it is NP-hard to enumerate all SCCs for a given network. Although most of the identified OCNs based on this set of SCCs are supported by multiple lines of evidence, more SCCs should be considered, if high-performance computing resources are available, in order to further improve the performance of OptiCon.

Supplementary Notes

Additional supporting evidence for identified optimal control nodes (OCNs) in liver cancer

APH1A encodes a subunit of gamma-secretase complex in the Notch signaling pathway, whose activation was demonstrated to promote the formation of hepatocellular carcinoma (HCC) *in vivo* [2]. Several gamma-secretase inhibitors are also in clinical trials for solid tumors, such as RO4929097 [3] and PF-03084014 [4]. MAPKAPK2 inhibitor, PHA-781089, was also reported to suppress HCC cell growth and induce HCC cell apoptosis [5].

In addition to the 10 OCNs that are known key regulators in HCC (Supplementary Table 4), we also identified five novel regulators, which may play a critical role in the progression of HCC. For instance, we found that *DNM1* knockout using the CRISPR-Cas9 technology can significantly inhibit the growth of SkHep1 liver cancer cells (Fig. 6b).

Additional supporting evidence for identified optimal control nodes (OCNs) in lung cancer

Targeting PARP1 can selectively kill lung adenocarcinoma (LUAD) cells with *ERCC1* or *PTEN* deficiency [6,7]. PARP1 is also documented in the Therapeutic Target Database as a target of the drug CEP-9722 (Phase 2 clinical trial) against non-small cell lung cancer. HIF1A inhibitor, PX-478, was reported to be effective against LUAD growth and metastasis *in vivo* [8]. Silencing of *PTP4A1* can decrease LUAD cell

invasiveness and enhance cell adhesion to reduce cancer metastasis [9]. Several PTP4A1 inhibitors have been discovered for cancer treatment [10,11]. *EGR1* and *EGLN2* are two known tumor suppressor genes in LUAD. Their overexpressing can suppress cancer cell proliferation and migration [12-14].

Besides the 13 OCNs that play critical roles in LUAD, we also identified 10 novel regulators, which may be important for LUAD development (Supplementary Table 4). For example, TAF12 is a key regulator involving in the *RAS*-induced malignant transformation of colon cancer cells [15]. Because *KRAS* is also an oncogene frequently mutated in LUAD [16] and *TAF12* is also significantly up-regulated in our expression data (fold-change = 1.5, *t*-test p-value = 8.2E-12), the pathogenic and therapeutic roles of TAF12 in LUAD should be further experimentally investigated.

Additional supporting evidence for identified optimal control nodes (OCNs) in breast cancer

PLK1 encodes a regulator of several stages during mitotic progression. Silencing *PLK1* can suppress proliferation and induce apoptosis of breast cancer cells [17-19]. Several PLK1 inhibitors are currently in clinical trials for advanced solid tumors [20,21]. EGFR is an effective therapeutic target for combination therapy of metastatic breast cancer [22-26]. *ESPL1* and *EGR1* are known oncogene [27-29] and tumor suppressor gene [30,31] of breast cancer, respectively. *SNAI1* is a cancer metastasis-promoting gene [32,33], while *RELN* and *SIK1* are two metastasis suppressor genes of breast cancer [34,35].

Evaluation of OptiCon predictions using existing drug synergy screening data

We used the drug synergy screening data by Friedman *et al.* [36] to evaluate the performance of OptiCon. The Friedman *et al.* study identified 1,220 synergistic drug pairs out of the 108×108 drug combinations in the melanoma cell lines SK-MEL-28 and LOXIMVI. 3,030 gene pairs in our input network are targets of these synergistic drug pairs, about 0.02% of the network. As the input to OptiCon, we downloaded gene expression data from Gene Expression Omnibus (GSE31909), which were generated using two melanoma cell lines SK-MEL-28 and LOXIMVI and two normal melanocyte lines, HEMn and HEMa. Each cell line has three replicates. Using this dataset, OptiCon identified 18 optimal control nodes (OCNs) and 105 synergistic OCN pairs (Supplementary Table 8). Among the OCNs, six (33%) are known cancer drug targets and two (NR1H3 and EPHA5) of them are primary targets of T0901317 and Dasatinib, respectively, which are two drugs in the 108-drug pool used in the Friedman *et al.* study [36]. NR1H3 and EPHA5 were predicted by OptiCon to have a synergistic interaction (multi-testing corrected p-value = 0.05) and their corresponding drugs T0901317 and Dasatinib were also validated to have a synergistic anti-cancer effect in both SK-MEL-28 and LOXIMVI cell lines based on the drug combination study [36].

It is also worth noting that another two identified OCNs, CDC25A and CCNB1, are key cell cycle regulators. CDC25A was reported to be directly phosphorylated by cyclinD-CDK4/6 complexes and its phosphorylation can be regulated by CDK inhibitors [37]. CCNB1 activity can also be modulated by CDK

inhibitors [38]. Thus, we regarded CDC25A and CCNB1 as the indirect targets of CDK inhibitors. Based on the drug combination data [36], CDK inhibitors were shown to have a synergistic interaction with either T0901317 or Dasatinib. Four gene pairs targeted by these two drugs (i.e. CDC25A & NR1H3, CDC25A & EPHA5, CCNB1 & NR1H3, and CCNB1 & EPHA5) were also predicted by OptiCon to be synergistic. Taken together, five synergistic gene pairs predicted by OptiCon (out of 105 pairs, 4.8%) are also targeted by pairs of drugs that have been shown to be synergistic by Friedman *et al.* [36]. The overlap between our predicted synergistic gene pairs and targets of synergistic drug pairs by Friedman *et al.* is statistically significant (hypergeometric test p-value = 1.4E-11).

Supplementary References

1 Kuhn, H. W. The Hungarian method for the assignment problem. *Naval Research Logistics* **2**, 83-97 (1955).

2 Villanueva, A. *et al.* Notch signaling is activated in human hepatocellular carcinoma and induces tumor formation in mice. *Gastroenterology* **143**, 1660-1669 (2012).

3 Tolcher, A. W. *et al.* Phase I study of RO4929097, a gamma secretase inhibitor of Notch signaling, in patients with refractory metastatic or locally advanced solid tumors. *Journal of Clinical Oncology* **30**, 2348-2353 (2012).

4 Wei, P. *et al.* Evaluation of selective γ-secretase inhibitor PF-03084014 for its antitumor efficacy and gastrointestinal safety to guide optimal clinical trial design. *Molecular Cancer Therapeutics* **9**, 1618-1628 (2010).

5 Tran, D. D. H. *et al.* Treatment with MAPKAP2 (MK2) inhibitor and DNA methylation inhibitor, 5-aza dC, synergistically triggers apoptosis in hepatocellular carcinoma (HCC) via tristetraprolin (TTP). *Cellular Signalling* **28**, 1872-1880 (2016).

6 Postel-Vinay, S. *et al.* A high-throughput screen identifies PARP1/2 inhibitors as a potential therapy for ERCC1-deficient non-small cell lung cancer. *Oncogene* **32**, 5377-5387 (2013).

7 Minami, D. *et al.* Synergistic effect of olaparib with combination of cisplatin on PTEN-deficient lung cancer cells. *Molecular Cancer Research* **11**, 140-148 (2013).

8 Jacoby, J. J. *et al.* Treatment with HIF-1α antagonist PX-478 inhibits progression and spread of orthotopic human small cell lung cancer and lung adenocarcinoma in mice. *Journal of Thoracic Oncology* **5**, 940-949 (2010).

9 Achiwa, H. & Lazo, J. S. PRL-1 tyrosine phosphatase regulates c-Src levels, adherence, and invasion in human lung cancer cells. *Cancer Research* **67**, 643-650 (2007).

10 Bai, Y. *et al.* Novel anticancer agents based on targeting the trimer interface of the PRL phosphatase. *Cancer Research* **76**, 4805-4815 (2016).

11 Jiang, Z.-X. & Zhang, Z.-Y. Targeting PTPs with small molecule inhibitors in

cancer treatment. *Cancer and Metastasis Reviews* **27**, 263-272 (2008).

12 Ferraro, B., Bepler, G., Sharma, S., Cantor, A. & Haura, E. B. EGR1 predicts PTEN and survival in patients with non–small-cell lung cancer. *Journal of Clinical Oncology* **23**, 1921-1926 (2005).

13 Zhang, H. *et al.* EGR1 decreases the malignancy of human non-small cell lung carcinoma by regulating KRT18 expression. *Scientific Reports* **4**, 5416 (2014).

14 Xie, X. *et al.* Over-expression of prolyl hydroxylase-1 blocks NF-κB-mediated cyclin D1 expression and proliferation in lung carcinoma cells. *Cancer Genetics* **207**, 188-194 (2014).

15 Voulgari, A. *et al.* TATA box-binding protein–associated factor 12 is important for RAS-induced transformation properties of colorectal cancer cells. *Molecular Cancer Research* **6**, 1071-1083 (2008).

16 Govindan, R. *et al.* Genomic landscape of non-small cell lung cancer in smokers and never-smokers. *Cell* **150**, 1121-1134 (2012).

17 Yao, Y.-D. *et al.* Targeted delivery of PLK1-siRNA by ScFv suppresses Her2+ breast cancer growth and metastasis. *Science Translational Medicine* **4**, 130ra148 (2012).

18 Spänkuch, B., Kurunci-Csacsko, E., Kaufmann, M. & Strebhardt, K. Rational combinations of siRNAs targeting Plk1 with breast cancer drugs. *Oncogene* **26**, 5793-5807 (2007).

19 Hu, K., Law, J. H., Fotovati, A. & Dunn, S. E. Small interfering RNA library screen identified polo-like kinase-1 (PLK1) as a potential therapeutic target for

breast cancer that uniquely eliminates tumor-initiating cells. *Breast Cancer Research* **14**, R22 (2012).

20 Strebhardt, K. & Ullrich, A. Targeting polo-like kinase 1 for cancer therapy. *Nature Reviews Cancer* **6**, 321-330 (2006).

21 Gutteridge, R. E. A., Ndiaye, M. A., Liu, X. & Ahmad, N. Plk1 inhibitors in cancer therapy: from laboratory to clinics. *Molecular Cancer Therapeutics* **15**, 1427-1435 (2016).

22 Masuda, H. *et al.* Role of epidermal growth factor receptor in breast cancer. *Breast Cancer Research and Treatment* **136**, 331-345 (2012).

23 Manupati, K. *et al.* Inhibiting epidermal growth factor receptor signalling potentiates mesenchymal -epithelial transition of their responsiveness to anticancer drugs. *The FEBS Journal* **284**, 1830-1854 (2017).

24 Foley, J. *et al.* EGFR signaling in breast cancer: bad to the bone. *Seminars in Cell & Developmental Biology* **21**, 951-960 (2010).

25 Lim, S.-O. *et al.* EGFR signaling enhances aerobic glycolysis in triple-negative breast cancer cells to promote tumor growth and immune escape. *Cancer Research* **76**, 1284-1296 (2016).

26 Ali, R. & Wendt, M. K. The paradoxical functions of EGFR during breast cancer progression. *Signal Transduction and Targeted Therapy* **2**, 16042 (2017).

27 Mukherjee, M. *et al.* MMTV-Espl1 transgenic mice develop aneuploid, estrogen receptor alpha (ERα)-positive mammary adenocarcinomas. *Oncogene* **33**,

5511-5522 (2014).

28 Zhang, N. *et al.* Overexpression of Separase induces aneuploidy and mammary tumorigenesis. *Proceedings of the National Academy of Sciences* **105**, 13033-13038 (2008).

29 Finetti, P. *et al.* ESPL1 is a candidate oncogene of luminal B breast cancers. *Breast Cancer Research and Treatment* **147**, 51-59 (2014).

30 Huang, R. P. *et al.* Decreased Egr-1 expression in human, mouse and rat mammary cells and tissues correlates with tumor formation. *International Journal of Cancer* **72**, 102-109 (1997).

31 Yang, M., Teng, W., Qu, Y., Wang, H. & Yuan, Q. Sulforaphene inhibits triple negative breast cancer through activating tumor suppressor Egr1. *Breast Cancer Research and Treatment* **158**, 277-286 (2016).

32 Zhou, B. P. *et al.* Dual regulation of Snail by GSK-3β-mediated phosphorylation in control of epithelial–mesenchymal transition. *Nature Cell Biology* **6**, 931-940 (2004).

33 Wang, Y., Shi, J., Chai, K., Ying, X. & P Zhou, B. The role of Snail in EMT and tumorigenesis. *Current Cancer Drug Targets* **13**, 963-972 (2013).

34 Stein, T. *et al.* Loss of reelin expression in breast cancer is epigenetically controlled and associated with poor prognosis. *The American Journal of Pathology* **177**, 2323-2333 (2010).

35 Cheng, H. *et al.* SIK1 couples LKB1 to p53-dependent anoikis and suppresses metastasis. *Science Signaling* **2**, ra35 (2009).

36 Friedman, A. A. *et al.* Landscape of targeted anti-cancer drug synergies in melanoma identifies a novel BRAF-VEGFR/PDGFR combination treatment. *PLoS ONE* **10**, e0140310 (2015).

37 Dozier, C. *et al.* CyclinD-CDK4/6 complexes phosphorylate CDC25A and regulate its stability. *Oncogene* **36**, 3781 (2017).

38 Gavet, O. & Pines, J. Activation of cyclin B1–Cdk1 synchronizes events in the nucleus and the cytoplasm at mitosis. *The Journal of Cell Biology* **189**, 247-259 (2010).
