# Supplementary material for: Optimal control nodes in disease-perturbed networks as targets for combination therapy
Source: Nat Commun. 2019 May 16;10:2180. doi: 10.1038/s41467-019-10215-y (PMC6522545; doi:10.1038/s41467-019-10215-y)
Supplement: Supplementary file 3 — Description of Additional Supplementary Files [file 41467_2019_10215_MOESM3_ESM.pdf]

Description of Additional Supplementay Files

Supplementary Data 1. Human gene regulatory network used in this study. Interactions were collected from three manually curated pathway databases: Reactome (1,597 pathways) [39], KEGG (195 pathways) [40], and NCI-Nature Pathway Interaction Database (745 pathways) [41]. The network consists of 5,959 genes and 108,281 regulatory interactions.

Supplementary Data 2. Cancer-type-specific recurrently mutated cancer genes documented in the Catalogue of Somatic Mutations in Cancer (COSMIC) database [42].

Supplementary Data 3. Experimentally derived cancer-type-specific synthetic lethal interactions documented in the Synthetic Lethality Database (SynLethDB) [43] and a published study [44] on genetic interaction screen using the CRISPR-Cas9 technology.

Supplementary Data 4. Supporting evidence for discovered optimal control nodes (OCNs) in three cancer types. HCC, hepatocellular carcinoma; LUAD, lung adenocarcinoma; BRCA, breast invasive carcinoma; Known cancer drug targets are obtained from the Therapeutic Target Database [45]. PMID, PubMed reference ID.

Supplementary Data 5. Identified synergistic optimal control node (OCN) pairs in three cancer types. P-values were adjusted for multiple testing using the method of Benjamini-Hochberg.

Supplementary Data 6. Supporting evidence for the role of crosstalk genes in therapy resistance. Literature evidence is presented for crosstalk genes controlled by both synergistic and non-synergistic OCN pairs.

Supplementary Data 7. sgRNA sequences for CRISPR-based growth assay used in this study.

Supplementary Data 8. Identified optimal control nodes (OCNs) and synergistic OCN pairs in melanoma.

Supplementary Table 9. Proteins associated with treatment side effect from the study [46].
